# Support for viral persistence in bats from age-specific serology and models of maternal immunity

**DOI:** 10.1038/s41598-018-22236-6

**Published:** 2018-03-01

**Authors:** Alison J. Peel, Kate S. Baker, David T. S. Hayman, Christopher C. Broder, Andrew A. Cunningham, Anthony R. Fooks, Romain Garnier, James L. N. Wood, Olivier Restif

**Affiliations:** 1 Disease Dynamics Unit, Department of Veterinary Medicine, University of Cambridge, Cambridge, CB3 0ES UK; 2 Institute of Zoology, Zoological Society of London, Regent’s Park, London, NW1 4RY UK; 3 Environmental Futures Research Institute, Griffith University, Brisbane, Queensland 4111 Australia; 4 Institute for Integrative Biology, University of Liverpool, Liverpool, L69 7ZB UK; 5 Animal and Plant Health Agency (APHA), Addlestone, Surrey, KT15 3NB UK; 6 Molecular Epidemiology and Public Health Laboratory, Hopkirk Research Institute, Massey University, Palmerston North, 4442 New Zealand; 7 Department of Microbiology and Immunology, Uniformed Services University, Bethesda, MD 20814-4799 USA

## Abstract

Spatiotemporally-localised prediction of virus emergence from wildlife requires focused studies on the ecology and immunology of reservoir hosts in their native habitat. Reliable predictions from mathematical models remain difficult in most systems due to a dearth of appropriate empirical data. Our goal was to study the circulation and immune dynamics of zoonotic viruses in bat populations and investigate the effects of maternally-derived and acquired immunity on viral persistence. Using rare age-specific serological data from wild-caught *Eidolon helvum* fruit bats as a case study, we estimated viral transmission parameters for a stochastic infection model. We estimated mean durations of around 6 months for maternally-derived immunity to Lagos bat virus and African henipavirus, whereas acquired immunity was long-lasting (Lagos bat virus: mean 12 years, henipavirus: mean 4 years). In the presence of a seasonal birth pulse, the effect of maternally-derived immunity on virus persistence within modelled bat populations was highly dependent on transmission characteristics. To explain previous reports of viral persistence within small natural and captive *E. helvum* populations, we hypothesise that some bats must experience prolonged infectious periods or within-host latency. By further elucidating plausible mechanisms of virus persistence in bat populations, we contribute to guidance of future field studies.

## Introduction

When a previously-unknown infectious disease emerges in human populations, wildlife species are often the focus of investigations aimed at identifying the natural reservoir host. However, this is just the first challenge; predicting, managing and preventing spillover of emerging infectious diseases to people and domestic animals depends on data, on reservoir host distribution, ecology, and immunology, as well as the mechanisms governing pathogen persistence within, and transmission among, its populations^1^. Due to the inherent challenges of studying wildlife and the diversity of potential hosts of zoonotic pathogens, such data are generally sparse.

Mathematical modelling approaches can circumvent some of these challenges and allow exploration of underlying processes, which can then be tested across a broad range of systems. For example, we recently investigated the importance of seasonal birth pulses and population immunity on viral persistence in closed populations across a diverse range of wild mammals^2^. By examining the critical community size (CCS, the threshold population size below which a virus is more likely to fade out than persist^3^), we showed that viruses are more likely to go extinct when the majority of the annual births take place within a short period (e.g. 1 month), compared with a species with year-round births. The effect on CCS of virus introduction into immune populations varied with the timing of the birth pulse relative to the initial epidemic peak: immunity either dampened epidemics and increased persistence (similar to the ‘priming for persistence’ phenomenon demonstrated by Pulliam *et al*.^4,5^), or resulted in higher rates of endemic fadeout and thereby decreased persistence^2^.

One factor rarely considered, but which might counteract the “boom and bust” virus dynamics generated by birth pulses, and thereby increase CCS, is maternal transfer of immunity^6–9^. Although maternally-derived antibodies (MatAb) have been reported in many vertebrate species (reviewed by ^6,10^, see also Supplementary Text 1), few studies have measured the persistence of those antibodies in wildlife or explored the effect of MatAb on viral dynamics (Supplementary Text 1). Compared with MatAb studies in domesticated animals^11^, challenges exist in model parameterisation for wildlife systems since pathogens are often endemic and affected by seasonal demography and MatAb persistence is largely unknown.

The simplifying assumptions often made in modelling studies (e.g. single, closed population of a single species), are analogous to the simplifications provided by empirical island studies. Logistical challenges aside, island populations therefore provide ideal natural experiments for studying host and disease ecology at a population level^12^. This is particularly the case for bats, where species traits such as nomadicism and fission-fusion population structures^13,14^, make it otherwise challenging to separate the dynamical effect of pathogen reintroduction into a study population from the transmission dynamics expected within a closed population. The population of African straw-coloured fruit bats (*Eidolon helvum*) on the remote Annobón island, Equatorial Guinea presents a rare opportunity to study viral dynamics in bat populations. This species is common and widely-distributed across continental sub-Saharan Africa and offshore islands, and recognised as a reservoir host for several potentially-zoonotic viruses across its range, including African bat henipavirus and Lagos bat virus (LBV, genus Lyssavirus)^15^. Located more than 180 km from the nearest island and 350 km from the African continent, the population of *E. helvum* bats on Annobón are both physically and genetically isolated^15^.

We have previously shown that persistence of the henipavirus Hendra virus was unlikely within single populations with the short infectious period demonstrated in experimentally infected animals^16^. Endemic circulation of Hendra virus in Australian flying foxes^17^, as well apparent persistence of henipaviruses in *E. helvum* on Annobón (estimated < 2500 bats) and in small captive colonies^18^ suggests that factors important for viral persistence in those systems were absent from the model^2^.

Here, we aim to elucidate fundamental processes governing viral dynamics in African bats, building on an extensive body of work on straw-coloured fruit bats (*Eidolon helvum*). We focus on two viruses for which *E. helvum* is a reservoir (LBV and African henipavirus), we look for evidence for the presence of MatAb in wild *E. helvum* and, uniquely, we use unique age-specific data to model waning rates of maternally- and infection- derived antibodies. We then focus specifically on population-level persistence of African henipavirus in the presence of MatAb, in both naive and non-naive populations, using the data to inform parameterisation of a stochastic seasonal birth model. We predict that the presence and increasing duration of MatAb will effectively reduce the ‘tightness’ of the birth pulse by dispersing the supply of susceptibles over a longer period. Applied to *E. helvum* bats in the isolated population on Annobón island, our results suggest that viral persistence would require long infectious periods or within-host viral latency, allowing us to narrow in on plausible mechanisms of virus persistence in bat populations and better understand infectious disease emergence.

## Methods

Sample collection methods, locations and serological analyses have been described elsewhere^13,15,19^, and the dataset is available online (Dryad Digital Repository: http://dx.doi.org/10.5061/dryad.2fp34). In brief, fieldwork was conducted with appropriate local permissions and Zoological Society of London Ethics Committee approval (WLE/0489 and WLE/0467) and all methods were performed in accordance with the relevant guidelines and regulations. Bats were caught in mist nets or samples were obtained from other research groups or from bats hunted for human consumption (Sample sizes are shown in Supplementary Table 1). Morphometric (forearm length and body mass) and demographic (age, sex, reproductive status) data were recorded. Criteria for assessing age, reproductive status and the phase in the reproductive cycle (months since the beginning of the previous birth pulse) are described in Peel *et al*.^19^.

Blood samples were collected from Ghana, Tanzania, Bioko, São Tomé, Príncipe and Annobón under manual restraint as described previously^19^. From bats that were hunted, or euthanased for associated virological studies or on welfare grounds (Ghana, Tanzania, São Tomé and Príncipe), upper canine teeth were extracted, air dried and processed histologically to allow ageing based on tooth cementum annuli (Matson’s laboratory, USA)^19,20^. The age of all wild-caught bats was estimated to the nearest month. Neonates (N, <2 months), juveniles (J, 2 –<6 months) and sexually immature individuals (SI, 6 –<24 months) were aged from body size, sexual development and knowledge of the birth pulse timing^13^. For adults (A, ≥2 years), the age in years from tooth cementum data was converted to months and added to the number of months since the previous birth pulse, to give a total age in months.

### Serological assays

While full genome sequence has documented the existence of African bat henipavirus^21^, it has not yet been isolated and cross-reactive serological assays must be used to detect its presence^22^. Antibodies against African henipaviruses were detected using a Nipah virus (NiV) Luminex microsphere binding assay^23^. Our use of the terms ‘henipavirus/henipaviruses’ is generic and represents the unknown cross-reactivity with this and other unknown African henipaviruses. Bayesian mixture models^22^ were used to determine a conservative cutoff threshold for seropositivity so that samples with median fluorescent intensity (MFI) readings above this cutoff were ≥99% likely to be true seropositives (MFI = 94.2, see^19^). Antibodies against LBV (LBV.NIG56-RV1) were detected using a mFAVN assay, using the LBVNig56 isolate, as previously described^19,24^. Titres were considered positive at IC100 endpoint reciprocal dilutions >1:9 (100% neutralisation of virus).

### Statistical analyses

All statistical analyses were performed with the R software^25^. Variations in LBV and henipavirus seroprevalences across sampling events were analysed, first using univariate comparisons and χ^2^ tests, followed by linear mixed-effects models, to characterise changes in seroprevalence as a function of bat demography, while accounting for among-site and among-year variation. Specifically, age category (N, J, SI, A), sex and reproductive status (whether adult females were pregnant or lactating) were fixed effects, and roost location (to the country-level) and year were explored as random effects (see Supplementary Text 2 for further details). For henipavirus data, *ln*(MFI) was the response variable. Following Zuur *et al*.^26^, generalised linear regression models were assessed against linear mixed-effects models with various random effect structures (using the glm function, and the lme function in the nlme package^27^, respectively). Selection of terms for deletion or inclusion (including two-way interactions) was based on Akaike’s information criterion (AIC). The optimal AIC model was tested for significant improvement (at the 5% level) over simpler models using ANOVAs to arrive at the minimum adequate (hereafter, ‘best’) model. For LBV data, we used the glmer function in the lme4 package^28^ with binary neutralization assay results as the response variable.

Correlation between serological results of dam and suckling pup pairs (n = 16 pairs) was assessed using Spearman’s rank correlation (for LBV mFAVN titres) and Pearson’s product-moment correlation (for henipavirus Luminex MFIs). A Wilcoxin signed rank test was used to compare the means of paired dam-pup titres. Dam-pup pair data are presented in Supplementary Table 2.

### Modelling of antibody dynamics

While the serostatus of an adult typically reflects past viral exposure and infection, serostatus in a neonate or juvenile bat may be due to the presence and subsequent waning of maternally-derived antibodies, a different and independent process. To explore signatures of waning antibodies in neonate and juvenile bats, and assess whether antibody waning also occurred subsequent to natural infection, a three-compartment susceptible-immune model (distinguishing maternally-derived from infection-acquired immunity) was created to predict age-specific seroprevalence as a function of four parameters:

*p*_*0*_ = the proportion of individuals seropositive at birth (estimated from neonate seroprevalence)

λ = the force of infection, that is, the rate at which susceptible individuals become infected and immune per year (assuming negligible infection-associated mortality)

*r*_*i*_ = the rate of antibody waning following natural infection-associated immunity

*r*_*m*_ = the rate of maternally-derived antibody waning following birth

We assume that the population is at steady state (no temporal variations in the numbers of individuals in each category) and that antibodies confer immunity to infection.and model serology as a function of age. For any age (*a*), let *S*(*a*), *M*(*a*) and *I*(*a*) be the proportions of bats that are respectively seronegative (susceptible to infection), seropositive with maternally derived immunity, or seropositive due to past or current infection. We describe the dynamics of the system with two independent compartmental sub-models, one for the cohorts of bats who were protected by maternal immunity at birth (proportion *p*_0_), and the other for the cohorts of bats who were not protected at birth (proportion 1− *p*_0_). Maternal immunity is lost at rate *r*_*m*_, and infection-induced immunity at rate *r*_*i*_. We assume endemic equilibrium, an age-independent force of infection *λ*, an age-independent death rate *d* and no infection-associated mortality (no clinical signs have been demonstrated in henipavirus-infected bats^16^, and death from LBV infection appears to be rare in this species^29^). At steady state the birth rate is equal to the death rate. Furthermore, because the death rate is age-independent and infection-independent, the equations for the proportions *S*(*a*), *I*(*a*), *M*(*a*) at a given age are independent of the death rate. This can be checked by writing the equations for the number of individuals in each category, and normalising by the population size

1 $$N(a)=N(0){e}^{-da}.$$

The model is governed by this linear system of ordinary differential equations:

2a $$S^{\prime} ={r}_{m}M(a)+{r}_{i}M(a)-\lambda S(a)$$

2b $$I^{\prime} =\lambda S(a)-\,{r}_{i}I(a)$$

2c $$M^{\prime} =-{r}_{m}M(a)$$

with boundary conditions *M*(0) = *p*_0_, *S*(0) = 1 − *p*_0_, *I*(0) = 0. This linear system of differential equations can be solved analytically, producing the following age distribution of seropositive individuals *P*(*a*) = *I*(*a*) + *M*(*a*):

3 $$P(a)=\frac{\lambda }{\lambda +\,{r}_{i}}\,[1-\,{e}^{-a(\lambda +{r}_{i})}]+\,{p}_{0}{e}^{-a{r}_{m}}[1-\,\frac{\lambda }{\lambda +\,{r}_{i}\,+\,{r}_{m}\,}(1-\,{e}^{-a(\lambda +{r}_{i}-{r}_{m})})]$$

The basic reproduction ratio of the infection, R_0_, is given by the inverse of the proportion of seronegative bats at a steady state (R_0_ = 1/S*), which can be expressed as a function of the four model parameters plus the bats’ natural death rate *d* by integrating over all ages:

4 $${R}_{0}=\frac{d+{r}_{m}}{d(1-{p}_{0})+{r}_{m}}(1+\frac{\lambda }{d+{r}_{i}}).$$

Assuming that a bat of age a has a probability *I*(*a*) of being seropositive, given the values of the four parameters of the model we can form the likelihood of a dataset formed by *N* pairs (*a*_*n*_, *x*_*n*_) where *a*_*n*_ is the age in years of bat *n* and *x*_*n*_ is its immunological status (1 for positive and 0 for negative), as follows:

5 $$L({p}_{0},\lambda ,{r}_{i},{r}_{m})=\prod _{n=1}^{N}I{({a}_{n})}^{{x}_{n}}{[1-I({a}_{n})]}^{1-{x}_{n}}$$

The model was fitted to a binary serology dataset for each virus (sample sizes shown in Supplementary Table 1) by maximising a binomial likelihood function with respect to its four parameters, using the powell^30^ optimisation package in R version 3. The model was compared to one in which acquired immunity was assumed to be lifelong (i.e. *r*_*i*_ = 0) using AIC. In order to produce confidence regions on the four parameters and R_0_, we generated 1000 bootstrap maximum likelihood estimates after randomly resampling each dataset with replacement. 95% confidence intervals were computed as the 2.5–97.5% interpercentile range from the set of bootstrap estimates for each parameter.

### Stochastic simulations of population-level virus dynamics

To explore patterns in population-level persistence of henipaviruses, we then ran simulations of a stochastic model incorporating maternally-derived immunity and waning immunity (MSIRS: Maternally immune – Susceptible – Infectious – Recovered – Susceptible). For henipaviruses, this infection cycle is justified by experimental evidence: Hendra virus excretion followed by recovery in the absence of clinical signs has been demonstrated in experimentally infected bats^16^; however, almost nothing is known about henipavirus infection and its outcomes in bats following natural exposure^1,31^. We chose not to simulate infection dynamics for Lagos Bat virus, because Lyssaviruses in bats generally follow more complex infection cycles^32–35^.

The demographic model structure was based on Peel *et al*.^2^, with an annual birth pulse modelled as a periodic Gaussian function and a density-independent death rate (*m*) was matched so that population size was stable inter annually. The birth pulse tightness parameter (*s*) was estimated from published *E. helvum* birth records^36,37^. Transmission was frequency-dependent. The main modification of our previous model was the addition of a proportion (ρ) of pups from immune dams being born with maternal antibodies (M), which decay at rate (η), and the loss of acquired immunity for recovered bats (at rate ζ). Transmission and transition rates are outlined in Supplementary Table 3. Parameter ranges were selected based on expectations for *E. helvum* from this and previous empirical studies (Table 1). At the start of simulations, five infectious individuals were introduced 3 months prior to the birth pulse peak to minimise the likelihood that MatAb from the previous birth pulse would be present in the population in simulations with population immunity. The simulations were run using the adaptivetau package^38^ in R, which implements Cao *et al*.’s adaptive tau-leap algorithm^39^. We imposed an upper limit on the size of the leap to ensure minute changes in birth rates between consecutive time steps. Critical community sizes (CCS, the population size at which infection is expected to persist for 10 years at least 50% of the time, conditional on successful invasion) were calculated by fitting a binomial generalised linear model to extinctions using the dose.p function in the MASS package^40^ in R. The R code is available on github: https://github.com/orestif/metapopulation/blob/master/MSIR/Models/.

**Table 1:** Parameter ranges explored in a henipavirus MSIRS model. *s = 14.3 represents 95% of births occurring within 3.2 months.

Parameter	Values explored	Reference
Lifespan (1 /*m*)	4.5 years	Hayman and Peel, 2016
Birth pulse tightness, s	14.3*	Hayman, 2015
Birth pulse timing, τ	0.25 years	
Average population size, Ν	400–102, 400	
R_0_	2.13	This study
Infectious period (IP, 1/γ)	10, 30, 60 days	Halpin *et al*.^16^; Plowright *et al*.^8^
Duration of maternal immunity (1/η)	0, 1, 2, 4, 6, 8 months	This study
Duration of acquired immunity (1/ζ)	50 months	This study
Population immunity (PI)	0–0.9	

### Data Availability

Data used in this study is available from the Dryad Digital Repository (http://dx.doi.org/10.5061/dryad.2fp34), with the exception of the dam – pup serology titres, which are provided in Supplementary Table 2.

## Results

**Figure 1:**
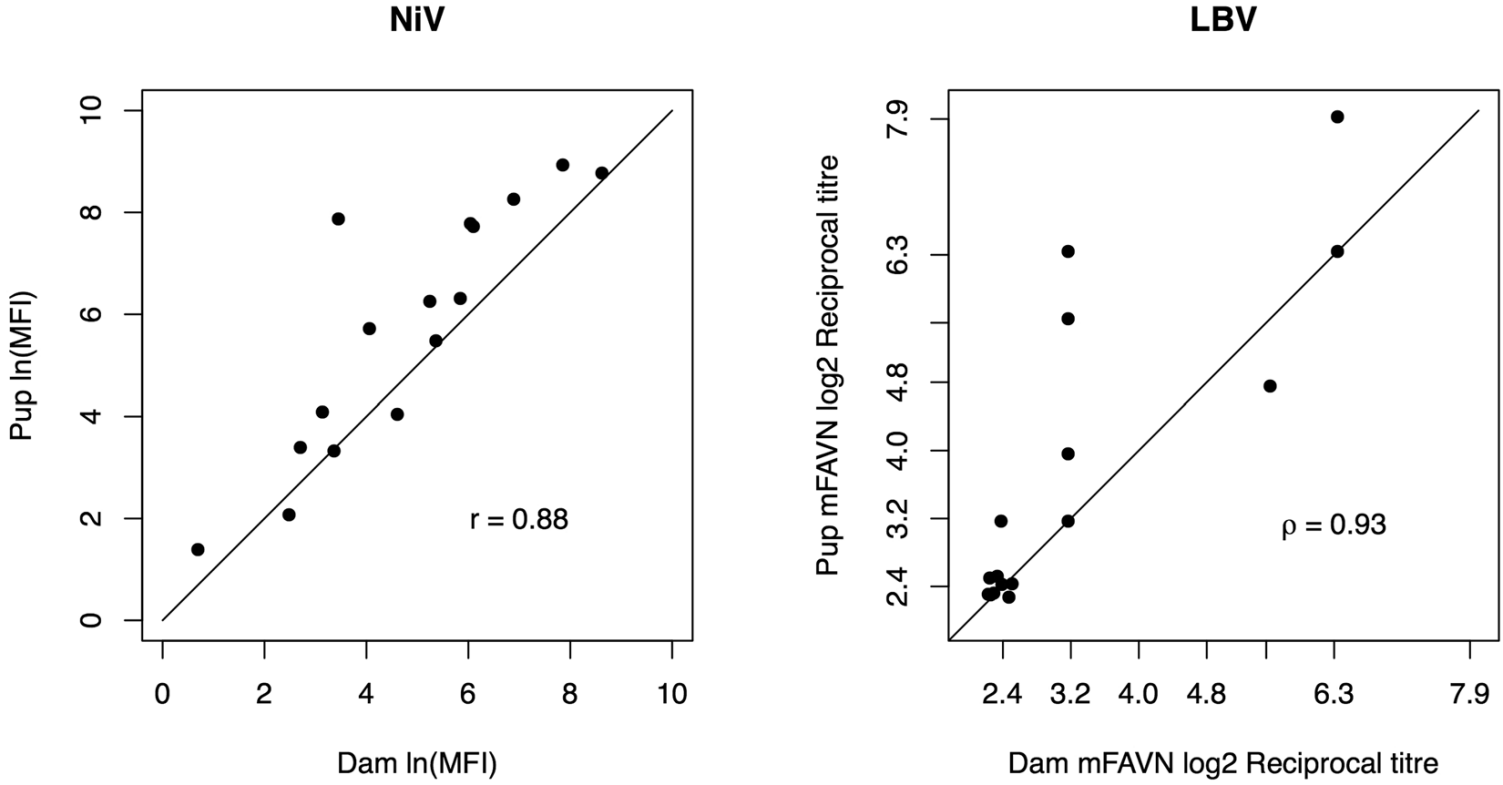
Correlation between NiV *ln*(MFI) values (left) and LBV mFAVN log_2_ reciprocal titres (right) of dam-pup pairs. The line represents equality of dam:pup titres. Points on the LBV plot have been jittered to demonstrate multiple overlaying points. Raw data are provided in Supplementary Table 2.

Evidence for maternal transfer of immunity was demonstrated by highly significant correlations for both LBV mFAVN reciprocal titre (ρ = 0.93, S = 50.676, p < 0.001) and NiV Luminex binding MFI (r = 0.88, t = 6.77, df = 14, p < 0.001) for 16 dam-pup pairs, in which the pups were suckling neonates (Fig. 1). Henipavirus titres were significantly higher in pups than dams (V = 11, p = 0.002).

**Figure 2:**
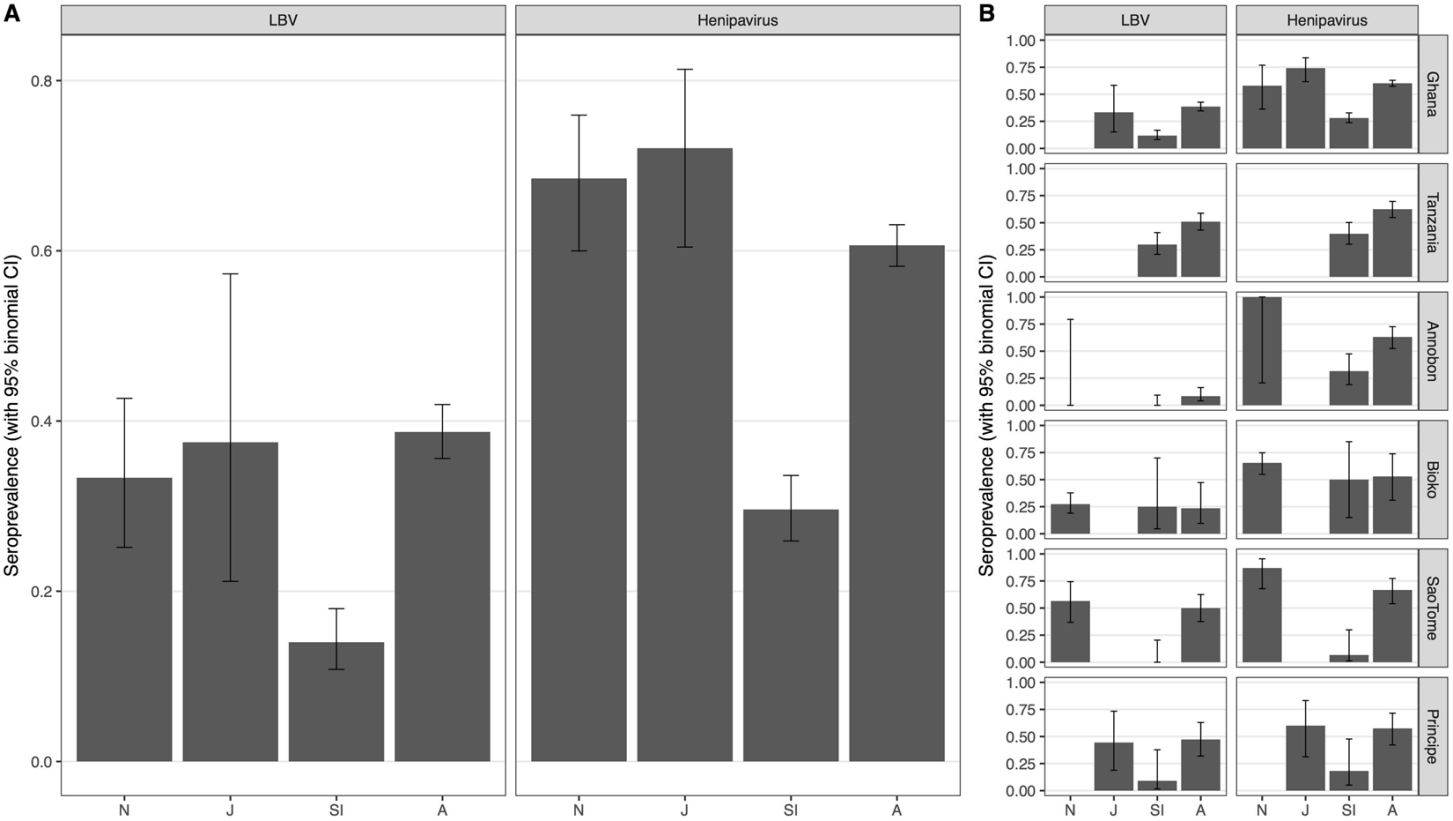
Henipavirus and Lagos Bat Virus (LBV) seroprevalence by age class (N: Neonate, J: Juvenile, SI: Sexually Immature, A: Adult) across all sites (**A**) and by roost site (**B**). Bars show binomial 95% confidence intervals. Sample sizes are shown in Supplementary Table 1.

LBV and henipavirus seroprevalence was highest among neonate, juvenile and adult age classes (Fig. 2). For henipaviruses, a fixed-effect generalised linear model was optimal to predict seroprevalence, with age class, sex and the interaction between age class and sex as explanatory variables (Supplementary Text 2), with no support for inclusion of random effects.

**Figure 3:**
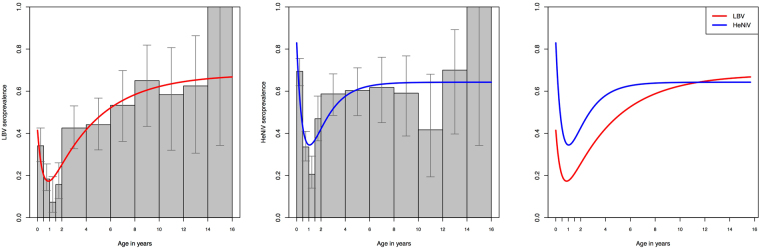
Predicted age-specific seroprevalences as determined by the waning immunity model. LBV (left) and henipavirus (right) predicted age-specific seroprevalence (red and blue line, respectively) overlying observed seroprevalences for all sampling locations combined (grey bars).

A mixed effect model for LBV was supported, with age class and sex as fixed effects and country as a random intercept, largely due to the significantly lower seroprevalence in Annobón (Fig. 2). Taking this into account, age class was the strongest determinant on seroprevalence (χ^2^(3) = 90.3, p < 0.01), with seroprevalence significantly lower in sexually immature bats compared with other age classes (Supplementary Text 2). Increasing seroprevalence with age (in years) across the entire dataset was supportive of endemic transmission (Fig. 3). Although age-specific data from teeth were not available from bats on Annobón, seroprevalence by extended age-classifications was also supportive of endemic transmission (Supplementary Fig. 1).

Age-specific seroprevalences, predicted as a function of neonate seroprevalence (*p*_*0*_), force of infection (λ), and two rates of waning antibodies (*r*_*i*_ and *r*_*m*_), showed a good fit to both LBV and henipavirus datasets, although the LBV fit was better for the older age groups (Fig. 3). Comparable parameter estimates were obtained when using data from all populations (results shown here), versus only from panmictic populations (Ghana, Tanzania, Bioko) or data only from genetically connected island populations (São Tomé and Príncipe). For both viruses, the AIC analysis supported the inclusion of waning acquired immunity (compared to a model with life-long immunity, *r*_*i*_ = 0), however for LBV this was marginal (ΔAIC = 2.3 *c.f*. henipaviruses: ΔAIC > 10), indicating that acquired immunity to LBV may be lifelong (Full model results shown in Supplementary Text 3). Given that the oldest individual in this study was 15 years, and the median age was 4.7 years, and the estimated mean duration of acquired immunity to LBV was 12 years (95% CI 5 – Infinity, Table 2), even the model with waning acquired immunity supports effective lifelong immunity to LBV. In contrast, the mean duration of acquired immunity to henipaviruses was just 4.1 years (95% CI 2.7–7.2, Table 2).

**Table 2:** Parameter estimates from the waning immunity model based on bat age in years, with 95% bootstrap confidence intervals: *p*_0_, the proportion of individuals seropositive at birth; *λ*, the force of infection, *r*_*i*_, the rate of antibody waning following natural infection; *r*_*m*_, the rate of antibody waning following birth.

	*R*_0_	*p*_0_	Rates (year^−1^)			Mean (in years)		
			Waning of maternal immunity *r*_*m*_	Force of infection *λ*	Waning of acquired immunity *r*_*i*_	Duration of maternal immunity 1/*r*_*m*_	Time to acquire immunity 1/*λ*	Duration of acquired immunity 1/*r*_*i*_
HeNiV	2.1 (1.9–2.3)	0.83 (0.73–0.93)	1.8 (1.4–2.2)	0.44 (0.33–0.55)	0.24 (0.14–0.38)	0.56 (0.45–0.70)	2.3 (1.8–3.0)	4.1 (2.7–7.2)
LBV	1.6 (1.5–1.8)	0.41 (0.31–0.55)	2.2 (1.4–3.3)	0.17 (0.12–0.24)	0.08 (0–0.19)	0.46 (0.31–0.68)	5.8 (4.2–7.9)	12 (5.2 – inf.^1^)

^1^lifelong immunity (no loss).

The rate of loss of maternally-derived immunity was comparable for both viruses, with a trough in seroprevalence around 11–12 months (Fig. 3) and estimates for the mean duration of maternal immunity of 5.2 months (95% CI 3.7–8.2) for LBV and 6.7 months (95% CI 5.4–8.4) for henipaviruses (Table 2).

Parameter estimates for neonate seroprevalence and force of infection were higher (with non-overlapping confidence intervals) for henipaviruses than for LBV (Table 2, Supplementary Fig. 4). On average, bats acquired immunity to LBV within 5 years 9 months of birth versus just 2 years 3 months for henipaviruses. Corresponding to the differences in force of infection, the R_0_ for henipavirus (2.1, 95% CI 1.9–2.3) was significantly higher than for LBV (1.6, 95% CI 1.5–1.8). These model-fitted R_0_ estimates were comparable to estimates calculated as 1/proportion seronegative (2.2 and 1.5 for henipavirus and LBV, respectively). We used the estimates from the fitted model as a basis for the simulations for the sake of coherence.

**Figure 4:**
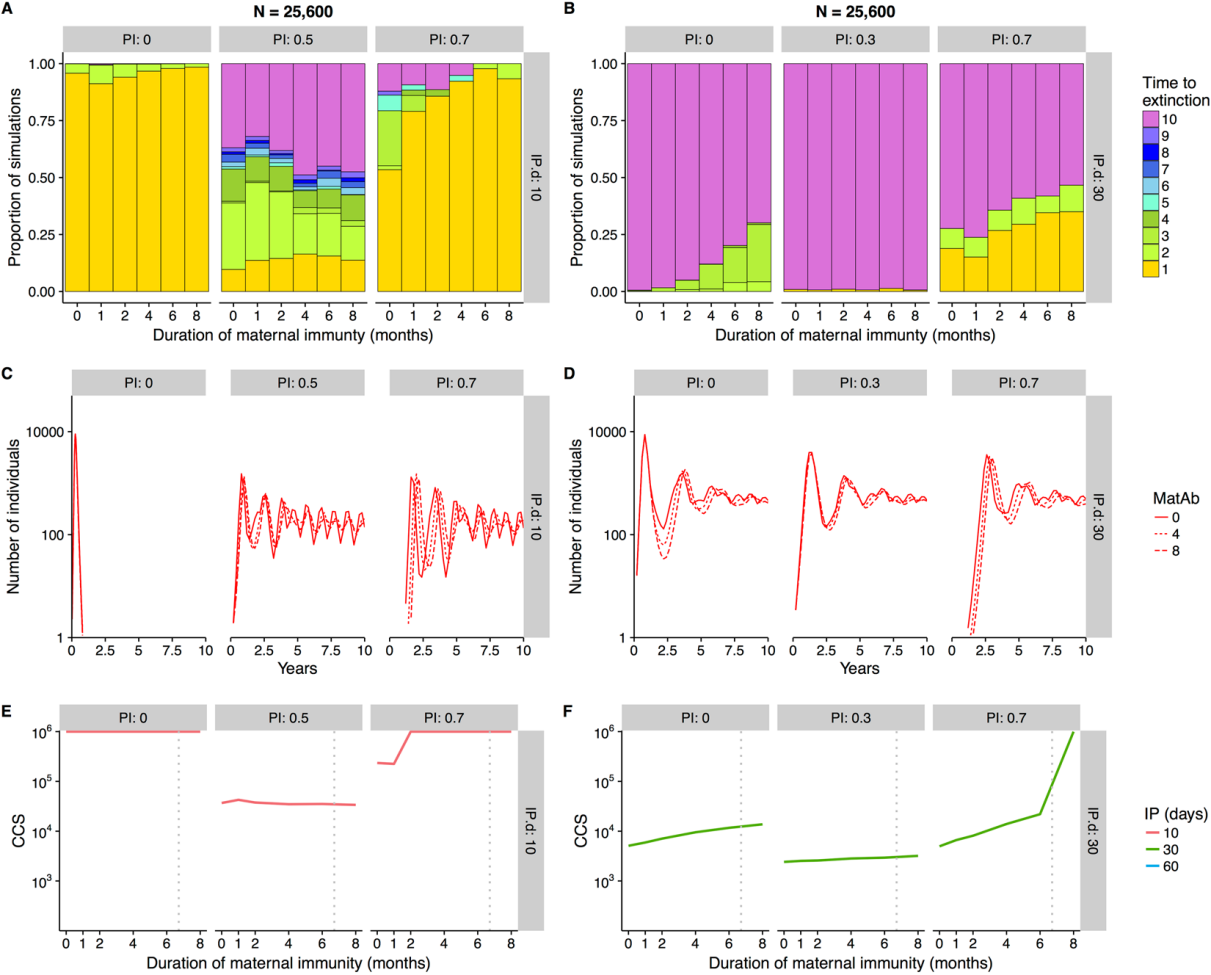
Effect of the duration of maternal antibody protection (in months, MAb), proportion of acquired population immunity (PI) and infectious period (left panels IP.d = 10 days, right panels, IP.d = 30 days) on: (**A** and **B**) persistence of infection, (**C** and **D**) number of infected individuals in a population, and (**E** and **F**) the population size for which successful of invasion and persistence of infection is more probable than not (critical community size, CCS). In A and B, stacked histograms show time to pathogen extinction (conditional on successful invasion) in series of 1000 stochastic simulations run for 10 years in a population size of 25,600 individuals. In deterministic simulations in C and D, population sizes are the same as in A and B. In E and F: grey dotted lines show the mean duration of maternal immunity, as calculated in the age-specific immunity model (henipaviruses = 6.7 months). For some sets of parameter values, probability of invasion was very low (Supplementary Fig. 5), resulting in low precision of the CCS estimate, as demonstrated by the jagged lines. Parameter values: mean lifespan = 4.5 years, *s* = 14.3, τ = 0.25, R_0_ = 2.13.

We developed an MSIRS model to explore the presence and rapid waning of maternal antibodies and long-lasting infection-induced antibodies. An invasion threshold occurs where the effective reproductive number (R_eff_) is less than one: here, given R_eff_ = R_0_(1 − PI) and R_0_ = 2.1, infection cannot spread if PI > 0.53^41^. As expected, the probability of successful invasion decreased as population immunity (PI) increased (according to the formula (1/R_eff_)^5^ when infection was seeded with 5 initial cases^42^, Supplementary Fig. 5). However, after conditioning on successful invasion, MatAb and population immunity had non-monotonic effects on viral persistence (Supplementary Figs 5–10. When R_eff_ > 1, successful invasion was followed by either persistent endemic cycles or ‘boom and bust’ epidemic fadeout. Epidemic fadeouts (time to extinction <2 years on Fig. 4A) typically occurred with short infectious periods in naïve populations, resulting in large CCS (Fig. 4A,C,E with PI = 0). Increasing population immunity (whilst keeping R_eff_ > 1) markedly decreased the CCS (Fig. 4E, PI = 0.5): high PI values dampened dynamics and allowed stochastic persistence through the post-epidemic trough in an increasing proportion of simulations (Supplementary Fig. 8). In these circumstances addition and increasing duration of MatAb had comparably little effect on dynamics and CCS (Fig. 4A,C,E, Supplementary Fig. 9).

Alternatively, when successful invasion led to more stable infection dynamics (e.g. with longer infectious periods, partially-immune populations; Fig. 4D), fadeout was either rare (Fig. 4B, PI = 0.3) or driven by stochastic endemic fadeout when prevalence was low (Fig. 4A, PI = 0.5). When the rate of endemic fadeout is low (pink colours in Fig. 4B), the effect of MatAb on CCS is again limited (e.g. nearly flat lines in Fig. 4F). However, where endemic fadeout is higher and centred around the 50% threshold (observed as a trickle of fadeouts each year, resulting in the full spectrum of colours observed in Fig. 4A, Supplementary Figs 6 & 8), increasing population immunity beyond the invasion threshold (so that R_eff_ < 1) further depletes susceptible individuals (Fig. 4A, PI = 0.7), resulting in low invasion success and return to a high CCS comparable with that in naïve populations (Fig. 4E, Supplementary Fig. 8). Additionally, at such high levels of population immunity, transmission is severely dampened. The fraction of introductions resulting in successful invasion propagate as slow, smouldering, low-amplitude transmission cycles^8^ until either endemic fadeout occurs or an epidemic eventually takes off (Supplementary Fig. 8, Supplementary Fig. 10). Here, the effect of the addition of MatAb is more apparent: transmission can smoulder for more than a year before initiating an epidemic. This long duration makes them more susceptible to annual smothering with the reduced influx of susceptible individuals during the birth pulse, which further dampens and slows transmission, leading to higher fadeout rates and higher CCS (Fig. 4B, PI = 0.7).

In general, trends are therefore dependent on unique combinations of demographic and transmission parameters. The diversity of the effects of acquired population immunity and maternally-derived immunity on CCS are shown in Supplementary Figs 5–10

## Discussion

To move beyond identification of broad geographic ‘hotspots’ of zoonotic viral emergence towards spatiotemporal prediction, management, and prevention of spillover at a local scale, we need to understand the finer scale drivers of disease dynamics in the reservoir host, and how seasonality and host immunity affect the timing of outbreak risk. Using empirical data from a wildlife host apparently capable of supporting endemic circulation of multiple viruses, we estimated relevant parameters and explored their effect on viral persistence in closed populations with seasonal births. We tested current alternative hypotheses on viral transmission dynamics in bats by applying these results to a natural system bearing strong resemblance to our model: a small, isolated island population, with an absence of alternative host species and immigration from other populations.

The existence of MatAb was supported by significant correlation of dam-pup assay titres, consistent with previous reports for bat rabies virus^43^, and for henipaviruses in captive bats^18^. Our observation of higher offspring titres than paired maternal titres is also consistent with those studies and with human studies (for example^44,45^). Furthermore, we estimated an average duration of maternally derived immunity to around 6 months for both viruses. This is in line with direct measurements by repeated sampling of captive-born *E. helvum* bats (6–7 months)^18,46^. The observed trough in seroprevalence in 1-year-old wild caught bats is consistent with captive *E. helvum*, where sexually immature bats were mostly found to seroconvert between 16–24 months^18^. Seroprevalence thereafter increased with age, with henipaviruses showing a significantly higher estimated force of infection than LBV, translating to a higher R_0_, an earlier age at first infection and more rapid rise in seroprevalence with age. Hayman *et al*.^24,47^ previously estimated R_0_ as 1.6 (95% CI 1.3–2.0) for both viruses from a single cross-sectional serosurvey of 59 bats in Ghana in 2007, based on the inverse of the proportion of seronegative individuals. With an expanded dataset (LBV: 1400 bats, henipaviruses: 2272 bats), our estimates using this method were comparable for LBV (95% CI 1.5–1.7) but greater for henipaviruses (2.1–2.3).

These empirical estimates enabled us to parameterise an infection model based solely on serological data from wild caught individuals, facilitating exploration of the critical community size for African henipaviruses in *E. helvum* bats as a function of their (as yet unknown) infectious periods. In most cases, the critical population size required for viral persistence was only minimally affected by the presence and duration of MatAb. The smaller proportion of the population capable of possessing MatAb also meant that the magnitude of the effect of these short-lived Ab was comparatively less than the presence of acquired immunity within the population as a whole.

Applying the predictions from our model to the isolated population of *E. helvum* on the remote island Annobón in the Gulf of Guinea, with an estimated size of <2500 bats, the persistence of henipavirus following a single introduction would require an infectious period of at least 40 days (Supplementary Figure 11). This estimate is considerably longer than current expectations: the infectious period for African henipaviruses are unknown but is ~7 days for the related Hendra virus in Australian fruit bats^16^. Alternatively, previous unsuccessful viral introductions, resulting in existing immunity within the population for the next introduction may play a role in henipaviral persistence in small populations^4,5^. Repeat migration events within the expected lifespan of immune bats, which would be required to establish persistent infection on Annobón, is not supported by the strong genetic differentiation and low number of mitochondrial haplotypes present in bats on the island^15^. Future age-specific serosurveys and genetic studies on Annobón are required to demonstrate seroconversion in bats born in the seven years since our first survey, confirm endemic circulation, and make further inferences on the likelihood of migrants contributing to persistence. Additionally, while preliminary explorations of the sensitivity of the MSIRS model to the estimated birth pulse duration indicated that even constant year-round births in Annobón would not significantly improve persistence (Supplementary Figure 11), further explorations of this across a more generic and wider range of parameter values would be valuable.

An alternative to long infectious periods and priming for persistence contributing to persistence of henipaviruses in small populations is the possibility that at least some individuals experience prolonged periods of viral latency or prolonged incubation periods. The similar antibody dynamics and waning estimates for LBV estimated parameters provide support for the need for at least some individuals to experience prolonged incubation periods. For LBV, a *Lyssavirus*, this would be consistent with expectations from field and laboratory studies of rabies virus-infected bats in the Americas, where individuals experience extended incubation periods between exposure until manifestation of clinical disease and infectiousness, for example^33,48^. A complexity not explored in our general model is that infection outcome may be dependent on the infection route and dose and that exposure of bats to rabies virus can induce seroconversion in the absence of clinical signs or becoming infectious^33^. Incubation periods and seroconversion in the absence of infectiousness have not yet been demonstrated for LBV in *E. helvum*, however future modelling studies would benefit from exploring this further. Persistent latent infection, with viral shedding during times of immunological stress has been suggested as contributing to henipavirus transmission dynamics^1,31^. Life-long latent infections with continuous or intermittent excretion would be expected to considerably lower the CCS. For some viruses in some animal species, persistent infections such as this may occur if infection happens during particular age ranges (e.g. foetal pestivirus infection in ruminants and camelids^49^ or neonatal arenavirus infection in wild rodents (*Mastomys natalensis*) result in persistent infection and long-lasting viral excretion, with or without antibody development^50^). Further models to explore the effect of latency on transmission dynamics and CCS are required.

We acknowledge that estimation of antibody waning rates (both for MatAb and infection-induced antibodies) with the method we used is challenged by accurate age estimation, irregular sampling periods and individual heterogeneity in initial titre. Environmental and seasonal effects on maternal physiology and immune status are expected to influence dam-pup MatAb transfer and subsequent waning times will be longer in individuals with high MatAb titres at birth e.g.^51^. The variable period over which waning of MatAb occurs (providing a ‘trickle’ of susceptible individuals), combined with the observation that bats born to seronegative dams are likely to become infected earlier than those born with MatAb^18^, could dampen the effect of the birth pulse on transmission and persistence. However, Baker *et al*.’s^18^ multi-year study in a small captive population demonstrated that seroconversions in adult females and in sexually immature bats all occurred within tight windows around pregnancy and breeding periods. It is unclear how henipaviruses are persisting in a small captive population between these periods. Similar windows of increased paramyxovirus transmission, apparently late pregnancy, have also been demonstrated in wild bat populations^52^.

Anamnestic responses (ongoing ‘boosting’ of antibody levels due to repeated infection) would complicate estimation of waning rates. These were supported by Baker *et al*.’s finding that^18^ 73% of positive henipavirus seroconversions in adult bats (>fourfold increase in antibody titre) represented antibody ‘boosting’, that is, occurred in already seropositive bats. Big brown bats (*E. fuscus*) experimentally infected with bat rabies virus^48^ developed antibodies which waned within 6 months in bats after a single exposure, but persisted for longer after each repeated exposure. Similarly, a study of wild *E. fuscus bats* demonstrated mean seroprevalence for anti-rabies virus antibodies was ~18% in wild adult females, yet their probability of seroconverting in a given year approached one^53^. Together with Turmelle *et al*.’s^48^ observation that some bats did not develop a detectable antibody response at all, these studies suggest that the proportion of bats within a natural population that have previously been exposed to rabies virus is likely to be considerably greater than the seroprevalence suggests. Similar findings have recently been reported in Egyptian rousette bats (*Rousettus aegyptiacus*) in an experimental setting, where Marburg virus infection led to viral excretion and seroconversion, followed by rapid antibody waning^54^. Re-exposure of these seronegative bats to Marburg virus resulted in rapid seroconversion in the absence of any evidence of viral replication or excretion^55^. This has implications for the parameterisation of viral dynamic models: we assume that anitbodies represent resistence to infection and that waning leads to an individual becoming susceptible. If infection leads to lifelong resistance to infection (and infectiousness) via other immune pathways, even in seronegative bats^55^, then critical community sizes would be larger than those estimated here. Further insights into bat immunology and longitudinal studies of individually isolated, naturally infected bats would be required to resolve this across multiple host-viral systems. Modelling SIR or SIRS dynamics with boosted antibody response in recovered individuals would also provide further insight.

Serosurveys have many benefits and a clear role in identifying reservoirs, however they present limitations for the inference of viral dynamics^1,56^. Availability of age-specific data was key to this study. In the absence of age-specific data (as is often the case in wildlife studies), exciting new approaches integrating laboratory studies and serological titres from cross-sectional field surveys show promise for the inference of time of infection, force of infection, and subsequently, population-level dynamics^57,58^. Yet, these methods rely on the existence of antibody titre data, measured repeatedly from multiple individuals. Generally this exists through experimental infection studies or, in some cases, in wild populations where high individual recapture rates are possible. For bat species, such as *E. helvum*, where the recapture of wild bats is exceptionally unlikely, and for emerging viruses, where experimental infections of highly pathogenic zoonoses are challenging and expensive, these data generally do not exist^59^. Additionally, a strong reliance on expected antibody dynamics inferred from model systems means these methods are “currently inaccessible for diseases with poorly understood or unpredictable immunological dynamics”^58^, such is the case for most bat viral infections^1,48^. Possibly due to unrealistic expectations that seronegative wild-caught bats have not previously been exposed to a particular virus, individual bat serological responses to experimental infections have been idiosyncratic. Despite these challenges, these methods are extremely promising and the additional insight that could be obtained from existing large cross-sectional serological datasets for emerging bat viruses provides further incentive for pursuing better understanding of bat immunology and immunological responses to specific bat pathogens.

## Conclusions

Elucidating bat viral dynamics faces many challenges, particularly that bat immunological responses are poorly understood and observed patterns in empirical data could equally result from multiple underlying processes and transmission mechanisms^1^. Here, we use age-specific serological data to obtain an unprecedented level of detail on bat viral dynamics and present the first quantification of viral antibody dynamics in wild African bats. While mechanisms like repeat introductions and metapopulation structure may drive viral dynamics in the large panmictic continental population of *E. helvum*, we have demonstrated that prolonged infectious periods or within-host latency are required to explain henipavirus persistence within small natural and captive populations^18,60^. Experimental and field studies exploring mechanisms of within-host viral persistence in bats should be a priority area for future research to inform our understanding of infectious disease emergence. Applicable across wildlife disease systems more broadly, we show that dynamic models informed by existing empirical data can help narrow-in on the most plausible competing hypothesis and guide future field studies^61^.

## Electronic supplementary material

Supplementary information
