# Supplementary material for: Support for viral persistence in bats from age-specific serology and models of maternal immunity
Source: Sci Rep. 2018 Mar 1;8:3859. doi: 10.1038/s41598-018-22236-6 (PMC5832774; doi:10.1038/s41598-018-22236-6)
Supplement: Supplementary file 1 — Supplementary information [file 41598_2018_22236_MOESM1_ESM.pdf]

SUPPLEMENTARY INFORMATION:

Support for viral persistence in bats from age-specific serology and models of maternal immunity

Alison J Peel*, Kate S Baker, David TS Hayman, Christopher C. Broder, Andrew A Cunningham, Anthony R Fooks, Romain Garnier, James LN Wood, and Olivier Restif

* alisonpeel@gmail.com

Contents:

Supplementary Text 1 2
Supplementary Text 2 4
Supplementary Text 3 17

Supplementary Table 1 3
Supplementary Table 2 15
Supplementary Table 3 15

Supplementary Figure 1 16
Supplementary Figure 2 18
Supplementary Figure 3 19
Supplementary Figure 4 21
Supplementary Figure 5 22
Supplementary Figure 6 23
Supplementary Figure 7 23
Supplementary Figure 8 24
Supplementary Figure 9 25
Supplementary Figure 10 25
Supplementary Figure 11 26

Supplementary Text 1:

MatAb have been directly or indirectly detected against henipaviruses in captive *E. helvum* (Baker et al., 2013), *Pteropus alecto* (Epstein et al., 2013), *P scapulatus* (Plowright et al., 2008), *P. poliocephalus*, *P. conspicillatus* (Field, 2005), and *P. vampyrus* (Sohayati et al., 2011), against Menangle virus in *P. poliocephalus* (Philbey et al., 2008), Marburg virus in *Rousettus aegyptiacus* (Amman et al., 2012), rabies virus in *Artibeus jamaicensis* (Price & Everard, 1977), *Eptesicus fuscus* (Shankar, Bowen, Davis, Rupprecht, & O'Shea, 2004) and *Tadarida brasiliensis mexicana* (Constantine, Tierkel, Kleckner, & Hawkins, 1968; Steece & Altenbach, 1989)) and in pups from bats vaccinated against canine distemper virus (*P. hypomelanus* (Epstein et al., 2013)). Four of these studies were longitudinal (Baker et al., 2013; Epstein et al., 2013; Shankar et al., 2004; Sohayati et al., 2011), demonstrating waning of MatAb over time in resampled pups.

Prior modelling studies exploring the effect of MatAb on viral dynamics have focused on their effects on the invasion threshold (Homwong, 2016; Pulliam et al., 2012), transmission rates (Allerson et al., 2013), timing of epidemics (Garnier, Gandon, Harding, & Boulinier, 2014; Kallio et al., 2010; Plowright et al., 2011; Wells et al., 2015), theoretical steady states (Bichara, Iggidr, & Sallet, 2013; Chapman, 2010) and on how interactions between MatAb and population fragmentation affect disease severity (Fouchet, Marchandeau, Bahi-Jaber, & Pontier, 2007). The effect of MatAb on population-level persistence has been explored in an intensive animal production system with a steady input of susceptible individuals: the presence of MatAb within piglets extended epidemic duration within cohorts, ultimately facilitating persistence within the total herd (Cador, Rose, Willem, & Andraud, 2016; Pulliam et al., 2012).

Supplementary Table 1: Sample sizes used in regression analyses and in age-specific modelling of antibody dynamics. The full dataset are available from the Dryad Digital Repository: http://dx.doi.org/10.5061/dryad.2fp34

Virus	Country	Neonate (N)	Juvenile (J)	Sexually Immature (SI)	Adult (A)	TOTAL SAMPLE SIZES FOR REGRESSION ANALYSES	Sample sizes used in age-specific modelling of antibody dynamics
Henipavirus	Annobón	1	0	38	84	123	39
	Bioko	0	84	4	17	105	104
	Ghana	9	58	381	1179	1637	347
	Príncipe	0	10	11	40	61	43
	São Tomé	23	0	15	60	98	94
	Tanzania	0	0	88	160	248	164
LBV	Annobón	1	0	37	83	121	38
	Bioko	84	0	4	17	105	104
	Ghana	0	15	220	557	792	183
	Príncipe	0	9	11	36	56	38
	São Tomé	23	0	15	58	96	92
	Tanzania	0	0	77	153	230	148

Supplementary Text 2:
Linear mixed-effect modelling approach and results

Following Zuur et al. [1], generalised linear regression models were assessed against linear mixed-effects models with various random effect structures, as described below

1. Henipaviruses

1. Start with a model containing all explanatory variables and interactions in the fixed part of the model

```
M1 = glm(log(outcome) ~ (Age * Sex.f) + Repro.f0)
```

2-6. Find the optimal random structure

To assess whether the mixed effects model is better than an ordinary regression model, we refit the latter using the gls function without the random intercept. The anova function was then be used to compare Akaike's information criterion (AIC) values.

```
M2 = gls(log(outcome) ~ (Age * Sex.f) + Repro.f0 ,na.action=na.omit)
M3 = lme(log(outcome) ~ (Age * Sex.f) + Repro.f0 ,random = list(Country.f=~1,
Year.f=~1),na.action=na.omit)
M4 = lme(log(outcome) ~ (Age * Sex.f) + Repro.f0 ,random =
~1|Country.f,na.action=na.omit)
M5 = lme(log(outcome) ~ (Age * Sex.f) + Repro.f0 ,random =
~1|Year.f,na.action=na.omit)
anova(M2,M3)

##    Model df      AIC      BIC    logLik   Test  L.Ratio p-value
## M2     1 12 7936.674 8005.012 -3956.337
## M3     2 14 7936.852 8016.580 -3954.426 1 vs 2 3.821458   0.148

anova(M4,M3)

##    Model df      AIC      BIC    logLik   Test  L.Ratio p-value
## M4     1 13 7937.524 8011.557 -3955.762
## M3     2 14 7936.852 8016.580 -3954.426 1 vs 2 2.672075  0.1021

anova(M5,M3)

##    Model df      AIC      BIC    logLik   Test  L.Ratio p-value
## M5     1 13 7938.276 8012.309 -3956.138
## M3     2 14 7936.852 8016.580 -3954.426 1 vs 2 3.423489  0.0643

anova(M2,M4)

##    Model df      AIC      BIC    logLik   Test  L.Ratio p-value
## M2     1 12 7936.674 8005.012 -3956.337
## M4     2 13 7937.524 8011.557 -3955.762 1 vs 2 1.149383  0.2837

anova(M2,M5)

##    Model df      AIC      BIC    logLik   Test   L.Ratio p-value
## M2     1 12 7936.674 8005.012 -3956.337
## M5     2 13 7938.276 8012.309 -3956.138 1 vs 2 0.3979692  0.5281
```

No significant difference with random effects - no evidence to favour mixed model

7-8. Find the optimal fixed structure

No support for mixed effect model, check for simpler model

```
step(M1)

## Start:  AIC=7913.51
## log(outcome) ~ (Age * Sex.f) + Repro.f0
##
##              Df Deviance    AIC
## - Repro.f0    3   4614.1 7911.4
## <none>            4606.0 7913.5
## - Age:Sex.f   3   4625.9 7917.0
##
## Step:  AIC=7911.41
## log(outcome) ~ Age + Sex.f + Age:Sex.f
##
##              Df Deviance    AIC
## <none>            4614.1 7911.4
## - Age:Sex.f   3   4628.2 7912.1

##
## Call:  glm(formula = log(outcome) ~ Age + Sex.f + Age:Sex.f)
##
## Coefficients:
##  (Intercept)          AgeJ         AgeSI          AgeA        Sex.fM
##     5.300809      0.233987     -1.316652     -0.008492     -0.091076
##  AgeJ:Sex.fM  AgeSI:Sex.fM   AgeA:Sex.fM
##    -0.141670      0.129311     -0.258250
##
## Degrees of Freedom: 2207 Total (i.e. Null);  2200 Residual
##   (1 observation deleted due to missingness)
## Null Deviance:       5120
## Residual Deviance: 4614  AIC: 7911
```

*Best model is log(outcome) ~ Age * Sex.f*

```
Mfinal = glm(log(outcome) ~ Age * Sex.f)
summary(Mfinal)

##
## Call:
## glm(formula = log(outcome) ~ Age * Sex.f)
##
## Deviance Residuals:
##    Min      1Q  Median      3Q     Max
## -4.313  -1.126   0.055   1.136   4.451
##
## Coefficients:
##              Estimate Std. Error t value Pr(>|t|)
## (Intercept)   5.300809   0.195277  27.145  < 2e-16 ***
## AgeJ          0.233987   0.315942   0.741    0.459
## AgeSI        -1.316652   0.216929  -6.070 1.51e-09 ***
## AgeA         -0.008492   0.208527  -0.041    0.968
## Sex.fM       -0.091076   0.260141  -0.350    0.726
## AgeJ:Sex.fM  -0.141670   0.437088  -0.324    0.746
## AgeSI:Sex.fM  0.129311   0.289199   0.447    0.655
## AgeA:Sex.fM  -0.258250   0.273769  -0.943    0.346
## ---
## Signif. codes:  0 '***' 0.001 '**' 0.01 '*' 0.05 '.' 0.1 ' ' 1
```

```
##
## (Dispersion parameter for gaussian family taken to be 2.097331)
##
##     Null deviance: 5120.4  on 2207  degrees of freedom
## Residual deviance: 4614.1  on 2200  degrees of freedom
##   (1 observation deleted due to missingness)
## AIC: 7911.4
##
## Number of Fisher Scoring iterations: 2
```

Summary

Significant effect of Age, Sex and Age:Sex in predicting henipavirus log(MFI)

2. Lagos Bat virus

1. Start with a model containing all explanatory variables and interactions in the fixed part of the model

```
M1 = glm(outcome ~ (Age * Sex.f) + Repro.f0, family = 'binomial')
```

2-6. Find the optimal random structure

To assess whether the mixed effects model is better than an ordinary regression model, we refit the latter using the gls function without the random intercept. The anova function was then be used to compare Akaike's information criterion (AIC) values.

```
M2 = glmer(outcome ~ (Age * Sex.f) + Repro.f0 + (1|Country.f) + (1|Year.f), family
= 'binomial')

## Warning in checkConv(attr(opt, "derivs"), opt$par, ctrl = control
## $checkConv, : Model failed to converge with max|grad| = 0.00171865 (tol =
## 0.001, component 1)
```

Model failed to converge.

Check singularity

The definition of singularity is that some of the constrained parameters of the random effects theta parameters are on the boundary (equal to zero, or very very close to zero, say <10^-6):

```
tt = getME(M2,'theta')
ll = getME(M2,'lower')
min(tt[ll==0])

## [1] 0.0005436179
```

Not a problem in this case

Restart

Try restarting from previous fit, with maximum number of iterations.

```
ss <- getME(M2,c("theta","fixef"))
M3 <- update(M2,start=ss,control=glmerControl(optCtrl=list(maxfun=2e4)))
```

```
## Warning in checkConv(attr(opt, "derivs"), opt$par, ctrl = control
## $checkConv, : Model failed to converge with max|grad| = 0.0193352 (tol =
## 0.001, component 1)
```

Still not converging.

Try a different optimizer

Try bobyqa for both phases – current GLMM default is bobyqa for first phase, Nelder-Mead for second phase.

```
M3 <- update(M2,start=ss,control=glmerControl(optimizer="bobyqa",optCtrl=list(maxf
un=2e5)))
summary(M3)

## Generalized linear mixed model fit by maximum likelihood (Laplace
##   Approximation) [glmerMod]
##  Family: binomial  ( logit )
## Formula:
## outcome ~ (Age * Sex.f) + Repro.f0 + (1 | Country.f) + (1 | Year.f)
## Control:
## glmerControl(optimizer = "bobyqa", optCtrl = list(maxfun = 2e+05))
##
##      AIC      BIC   logLik deviance df.resid
##   1631.7   1699.8   -802.8   1605.7     1386
##
## Scaled residuals:
##     Min      1Q  Median      3Q     Max
## -1.3304 -0.7484 -0.3884  1.0300  3.2786
##
## Random effects:
##  Groups    Name        Variance Std.Dev.
##  Country.f (Intercept) 0.73765  0.8589
##  Year.f    (Intercept) 0.02234  0.1495
## Number of obs: 1399, groups:  Country.f, 6; Year.f, 5
##
## Fixed effects:
##              Estimate Std. Error z value Pr(>|z|)
## (Intercept)  -0.22660    0.55019  -0.412 0.680445
## AgeJ         -0.82733    0.83718  -0.988 0.323040
## AgeSI        -1.80447    0.50835  -3.550 0.000386 ***
## AgeA          0.07757    0.52877   0.147 0.883368
## Sex.fM       -0.54356    0.42329  -1.284 0.199099
## Repro.f0NR   -0.82223    0.52262  -1.573 0.115656
## Repro.f0P    -0.08064    0.33047  -0.244 0.807218
## Repro.f0L    -0.71958    0.48860  -1.473 0.140823
## AgeJ:Sex.fM   1.05628    0.97983   1.078 0.281023
## AgeSI:Sex.fM  0.61482    0.52472   1.172 0.241317
## AgeA:Sex.fM   0.04459    0.50440   0.088 0.929551
## ---
## Signif. codes:  0 '***' 0.001 '**' 0.01 '*' 0.05 '.' 0.1 ' ' 1
##
## Correlation of Fixed Effects:
##             (Intr) AgeJ   AgeSI  AgeA   Sex.fM Rp.0NR Rpr.0P Rpr.0L AJ:S.M
## AgeJ        -0.384
## AgeSI       -0.614  0.484
## AgeA        -0.628  0.469  0.781
## Sex.fM      -0.395  0.262  0.433  0.416
## Repro.f0NR   0.000  0.009 -0.058 -0.281  0.001
```

```
## Repro.f0P    0.066  0.000 -0.026 -0.398 -0.002  0.362
## Repro.f0L    0.190 -0.169 -0.289 -0.543  0.000  0.312  0.417
## AgeJ:Sex.fM  0.168 -0.699 -0.189 -0.182 -0.432  0.013  0.001  0.003
## AgeSI:Sx.fM  0.326 -0.215 -0.557 -0.358 -0.807  0.015  0.045  0.020  0.349
## AgeA:Sex.fM  0.365 -0.224 -0.387 -0.618 -0.840  0.243  0.415  0.285  0.363
##             ASI:S.
## AgeJ
## AgeSI
## AgeA
## Sex.fM
## Repro.f0NR
## Repro.f0P
## Repro.f0L
## AgeJ:Sex.fM
## AgeSI:Sx.fM
## AgeA:Sex.fM  0.696
```

Model converged.

The Random effects and Std Dev columns provide a measure of how much variability in the log(MFI) is due to Year and Country (the two random effects). Country has more variability than Year.

Compare model with Country and Year as random effects versus just Country alone:

```
M4 = glmer(outcome ~ (Age * Sex.f) + Repro.f0 + (1|Country.f), family = 'binomial'
)

## Warning in checkConv(attr(opt, "derivs"), opt$par, ctrl = control
## $checkConv, : Model failed to converge with max|grad| = 0.00116531 (tol =
## 0.001, component 1)

ss <- getME(M4,c("theta","fixef"))
M5 <- update(M4,start=ss,control=glmerControl(optCtrl=list(maxfun=2e4)))
summary(M5)

## Generalized linear mixed model fit by maximum likelihood (Laplace
##   Approximation) [glmerMod]
##  Family: binomial  ( logit )
## Formula: outcome ~ (Age * Sex.f) + Repro.f0 + (1 | Country.f)
## Control: glmerControl(optCtrl = list(maxfun = 20000))
##
##      AIC      BIC   logLik deviance df.resid
##   1629.7   1692.6   -802.8   1605.7     1387
##
## Scaled residuals:
##     Min      1Q  Median      3Q     Max
## -1.3447 -0.7476 -0.3871  1.0325  3.2816
##
## Random effects:
##  Groups    Name        Variance Std.Dev.
##  Country.f (Intercept) 0.6989   0.836
## Number of obs: 1399, groups:  Country.f, 6
##
## Fixed effects:
##                Estimate Std. Error z value Pr(>|z|)
## (Intercept)    -0.25807    0.51968  -0.497 0.619482
## AgeJ           -0.82280    0.83581  -0.984 0.324897
## AgeSI          -1.80534    0.50719  -3.560 0.000372 ***
```

```
## AgeA          0.09540    0.52118   0.183 0.854754
## Sex.fM       -0.54309    0.42318  -1.283 0.199371
## Repro.f0NR   -0.82987    0.52068  -1.594 0.110975
## Repro.f0P    -0.11059    0.30999  -0.357 0.721288
## Repro.f0L    -0.73257    0.48396  -1.514 0.130104
## AgeJ:Sex.fM   1.05586    0.97985   1.078 0.281223
## AgeSI:Sex.fM  0.61241    0.52442   1.168 0.242895
## AgeA:Sex.fM   0.02832    0.49851   0.057 0.954695
## ---
## Signif. codes:  0 '***' 0.001 '**' 0.01 '*' 0.05 '.' 0.1 ' ' 1
##
## Correlation of Fixed Effects:
##              (Intr) AgeJ   AgeSI  AgeA   Sex.fM Rp.0NR Rpr.0P Rpr.0L AJ:S.M
## AgeJ         -0.404
## AgeSI        -0.649  0.483
## AgeA         -0.632  0.476  0.792
## Sex.fM       -0.417  0.263  0.433  0.421
## Repro.f0NR   -0.017  0.007 -0.059 -0.272  0.001
## Repro.f0P    -0.014 -0.003 -0.032 -0.374 -0.001  0.359
## Repro.f0L     0.171 -0.172 -0.291 -0.533  0.001  0.304  0.402
## AgeJ:Sex.fM   0.178 -0.700 -0.189 -0.185 -0.432  0.013  0.001  0.004
## AgeSI:Sx.fM   0.340 -0.216 -0.558 -0.360 -0.807  0.013  0.040  0.017  0.349
## AgeA:Sex.fM   0.356 -0.230 -0.394 -0.610 -0.849  0.233  0.393  0.271  0.368
##              ASI:S.
## AgeJ
## AgeSI
## AgeA
## Sex.fM
## Repro.f0NR
## Repro.f0P
## Repro.f0L
## AgeJ:Sex.fM
## AgeSI:Sx.fM
## AgeA:Sex.fM   0.701
```

```
anova(M5,M3)
```

```
## Data: NULL
## Models:
## M5: outcome ~ (Age * Sex.f) + Repro.f0 + (1 | Country.f)
## M3: outcome ~ (Age * Sex.f) + Repro.f0 + (1 | Country.f) + (1 | Year.f)
##    Df    AIC    BIC  logLik deviance  Chisq Chi Df Pr(>Chisq)
## M5 12 1629.7 1692.6 -802.84   1605.7
## M3 13 1631.7 1699.8 -802.84   1605.7 0.0134      1     0.9079
```

Model with Country and Year as random effects has a higher AIC than just Country alone, but not significantly so. This is expected, given the very low seroprevalence in Annobón).

Compare model with Country and Year as random effects to model with just year alone:

```
M6 = glmer(outcome ~ (Age * Sex.f) + Repro.f0 + (1|Year.f), family = 'binomial')
summary(M6)
```

```
## Generalized linear mixed model fit by maximum likelihood (Laplace
##   Approximation) [glmerMod]
##  Family: binomial  ( logit )
## Formula: outcome ~ (Age * Sex.f) + Repro.f0 + (1 | Year.f)
```

```
##
##      AIC      BIC   logLik deviance df.resid
##   1682.5   1745.4   -829.2   1658.5     1387
##
## Scaled residuals:
##     Min      1Q  Median      3Q     Max
## -1.1222 -0.7863 -0.4237  1.1598  2.8144
##
## Random effects:
##  Groups Name        Variance Std.Dev.
##  Year.f (Intercept) 0.0992   0.315
## Number of obs: 1399, groups:  Year.f, 5
##
## Fixed effects:
##               Estimate Std. Error z value Pr(>|z|)
## (Intercept)  -0.568385   0.347896  -1.634 0.102306
## AgeJ         -0.390388   0.754569  -0.517 0.604901
## AgeSI        -1.391098   0.381356  -3.648 0.000265 ***
## AgeA          0.459172   0.406728   1.129 0.258923
## Sex.fM       -0.479087   0.412266  -1.162 0.245202
## Repro.f0NR   -0.813661   0.493672  -1.648 0.099316 .
## Repro.f0P    -0.077371   0.308821  -0.251 0.802173
## Repro.f0L    -0.616037   0.425236  -1.449 0.147423
## AgeJ:Sex.fM   1.036188   0.968602   1.070 0.284720
## AgeSI:Sex.fM  0.618500   0.513210   1.205 0.228142
## AgeA:Sex.fM   0.004894   0.491438   0.010 0.992055
## ---
## Signif. codes:  0 '***' 0.001 '**' 0.01 '*' 0.05 '.' 0.1 ' ' 1
##
## Correlation of Fixed Effects:
##              (Intr) AgeJ   AgeSI  AgeA   Sex.fM Rp.0NR Rpr.0P Rpr.0L AJ:S.M
## AgeJ         -0.348
## AgeSI        -0.696  0.322
## AgeA         -0.688  0.307  0.644
## Sex.fM       -0.574  0.264  0.524  0.491
## Repro.f0NR   -0.005 -0.024 -0.077 -0.372  0.000
## Repro.f0P     0.033 -0.006 -0.075 -0.532  0.000  0.423
## Repro.f0L     0.065 -0.035 -0.101 -0.478  0.000  0.393  0.519
## AgeJ:Sex.fM   0.249 -0.766 -0.227 -0.214 -0.425  0.003  0.001  0.005
## AgeSI:Sx.fM   0.462 -0.212 -0.690 -0.415 -0.803  0.017  0.040  0.020  0.341
## AgeA:Sex.fM   0.498 -0.224 -0.473 -0.751 -0.839  0.266  0.418  0.330  0.357
##              ASI:S.
## AgeJ
## AgeSI
## AgeA
## Sex.fM
## Repro.f0NR
## Repro.f0P
## Repro.f0L
## AgeJ:Sex.fM
## AgeSI:Sx.fM
## AgeA:Sex.fM   0.691
```

```
anova(M6,M3)
```

```
## Data: NULL
## Models:
## M6: outcome ~ (Age * Sex.f) + Repro.f0 + (1 | Year.f)
## M3: outcome ~ (Age * Sex.f) + Repro.f0 + (1 | Country.f) + (1 | Year.f)
```

```
##    Df    AIC    BIC  logLik deviance  Chisq Chi Df Pr(>Chisq)
## M6 12 1682.5 1745.4 -829.23   1658.5
## M3 13 1631.7 1699.8 -802.84   1605.7 52.789      1  3.714e-13 ***
## ---
## Signif. codes:  0 '***' 0.001 '**' 0.01 '*' 0.05 '.' 0.1 ' ' 1
```

Model with Country and Year as random effects is much better than just year alone.

Best random effects structure is + (1|Country.f)

7-8. Find the optimal fixed structure

Try removing Reproductive status as a fixed effect:

```
M7 = glmer(outcome ~ (Age * Sex.f) + (1|Country.f), family = 'binomial')
anova(M5,M7)

## Data: NULL
## Models:
## M7: outcome ~ (Age * Sex.f) + (1 | Country.f)
## M5: outcome ~ (Age * Sex.f) + Repro.f0 + (1 | Country.f)
##    Df    AIC    BIC  logLik deviance  Chisq Chi Df Pr(>Chisq)
## M7  9 1627.8 1675.0 -804.92   1609.8
## M5 12 1629.7 1692.6 -802.84   1605.7 4.1569      3      0.245
```

Model 7 (without reproductive status) is lower AIC. Although it is not significantly improved, it is a simpler model, so go with that.

Try removing Age:Sex interaction as a fixed effect:

```
M8 = glmer(formula=outcome ~ (Age + Sex.f) + (1|Country.f), family = 'binomial',na
.action = na.omit)
anova(M7,M8)

## Data: NULL
## Models:
## M8: outcome ~ (Age + Sex.f) + (1 | Country.f)
## M7: outcome ~ (Age * Sex.f) + (1 | Country.f)
##    Df    AIC    BIC  logLik deviance  Chisq Chi Df Pr(>Chisq)
## M8  6 1624.4 1655.8 -806.18   1612.4
## M7  9 1627.8 1675.0 -804.92   1609.8 2.5105      3     0.4734
```

Model 8 (without Age:Sex interaction) is lower AIC. Although it is not significantly improved, it is a simpler model, so go with that.

Try removing Sex as a fixed effect:

```
M9 = glmer(formula = outcome ~ Age  + (1|Country.f), family = 'binomial',na.action
= na.omit)
summary(M9)

## Generalized linear mixed model fit by maximum likelihood (Laplace
##   Approximation) [glmerMod]
##  Family: binomial  ( logit )
## Formula: outcome ~ Age + (1 | Country.f)
##
##      AIC      BIC   logLik deviance df.resid
##   1625.7   1651.9   -807.8   1615.7     1395
##
```

```
## Scaled residuals:
##     Min      1Q  Median      3Q     Max
## -1.0860 -0.7843 -0.3842  1.0764  3.5531
##
## Random effects:
##  Groups    Name        Variance Std.Dev.
##  Country.f (Intercept) 0.696    0.8343
## Number of obs: 1400, groups:  Country.f, 6
##
## Fixed effects:
##             Estimate Std. Error z value Pr(>|z|)
## (Intercept)  -0.4827     0.4567  -1.057    0.291
## AgeJ         -0.3188     0.5654  -0.564    0.573
## AgeSI        -1.6253     0.3867  -4.203 2.63e-05 ***
## AgeA         -0.1979     0.3568  -0.555    0.579
## ---
## Signif. codes:  0 '***' 0.001 '**' 0.01 '*' 0.05 '.' 0.1 ' ' 1
##
## Correlation of Fixed Effects:
##       (Intr) AgeJ   AgeSI
## AgeJ  -0.419
## AgeSI -0.588  0.582
## AgeA  -0.631  0.625  0.899
```

```
anova(M9,M8)
Data: NULL
Models:
M9: outcome ~ Age + (1 | Country.f)
M8: outcome ~ (Age + Sex.f) + (1 | Country.f)
   Df    AIC    BIC  logLik deviance  Chisq Chi Df Pr(>Chisq)
M9  5 1625.7 1651.9 -807.83   1615.7
M8  6 1624.7 1656.1 -806.32   1612.7 3.0205      1    0.08222 .
---
Signif. codes:  0 ‘***’ 0.001 ‘**’ 0.01 ‘*’ 0.05 ‘.’ 0.1 ‘ ’ 1
```

Model 8 (including Sex as a fixed effect) has a slightly lower AIC than with Sex removed, but this is not significant. Model 9 is the simpler model though.

Model 10: Try removing Age as a fixed effect (with Sex included), and compare with model 8:

```
M10 = glmer(formula = outcome ~ Sex.f  + (1|Country.f), family = 'binomial',na.act
ion = na.omit)
summary(M10)

## Generalized linear mixed model fit by maximum likelihood (Laplace
##   Approximation) [glmerMod]
##  Family: binomial  ( logit )
## Formula: outcome ~ Sex.f + (1 | Country.f)
##
##      AIC      BIC   logLik deviance df.resid
##   1708.6   1724.4   -851.3   1702.6     1396
##
## Scaled residuals:
##     Min      1Q  Median      3Q     Max
## -0.8825 -0.6753 -0.6691  1.1436  3.4258
##
## Random effects:
##  Groups    Name        Variance Std.Dev.
```

```
##   Country.f (Intercept) 0.6265   0.7915
## Number of obs: 1399, groups:  Country.f, 6
##
## Fixed effects:
##             Estimate Std. Error z value Pr(>|z|)
## (Intercept) -0.88094    0.34412  -2.560   0.0105 *
## Sex.fM      -0.01831    0.12536  -0.146   0.8839
## ---
## Signif. codes:  0 '***' 0.001 '**' 0.01 '*' 0.05 '.' 0.1 ' ' 1
##
## Correlation of Fixed Effects:
##        (Intr)
## Sex.fM -0.213
```

```
anova(M10,M8)
```

```
## Data: NULL
## Models:
## M10: outcome ~ Sex.f + (1 | Country.f)
## M8: outcome ~ (Age + Sex.f) + (1 | Country.f)
##     Df    AIC    BIC  logLik deviance  Chisq Chi Df Pr(>Chisq)
## M10  3 1708.6 1724.4 -851.31   1702.6
## M8   6 1624.4 1655.8 -806.18   1612.4 90.269      3  < 2.2e-16 ***
## ---
## Signif. codes:  0 '***' 0.001 '**' 0.01 '*' 0.05 '.' 0.1 ' ' 1
```

Model 8 (Age, but not Sex as a fixed effect) has a significantly lower AIC than Model 10.

AIC cannot distinguish between:

outcome ~ Age + Sex.f + (1|Country.f)

outcome ~ Age + (1|Country.f)

```
summary(M8)
```

```
## Generalized linear mixed model fit by maximum likelihood (Laplace
##   Approximation) [glmerMod]
##  Family: binomial  ( logit )
## Formula: outcome ~ (Age + Sex.f) + (1 | Country.f)
##
##      AIC      BIC   logLik deviance df.resid
##   1624.4   1655.8   -806.2   1612.4     1393
##
## Scaled residuals:
##     Min      1Q  Median      3Q     Max
## -1.1833 -0.7631 -0.4103  1.0202  3.8986
##
## Random effects:
##  Groups    Name        Variance Std.Dev.
##  Country.f (Intercept) 0.7096   0.8424
## Number of obs: 1399, groups:  Country.f, 6
##
## Fixed effects:
##             Estimate Std. Error z value Pr(>|z|)
## (Intercept)  -0.3135     0.4699  -0.667   0.5046
## AgeJ         -0.3806     0.5689  -0.669   0.5034
## AgeSI        -1.7000     0.3925  -4.331 1.48e-05 ***
## AgeA         -0.2259     0.3607  -0.626   0.5311
## Sex.fM       -0.2328     0.1329  -1.751   0.0799 .
```

```
## ---
## Signif. codes:  0 '***' 0.001 '**' 0.01 '*' 0.05 '.' 0.1 ' ' 1
##
## Correlation of Fixed Effects:
##        (Intr) AgeJ   AgeSI  AgeA
## AgeJ   -0.423
## AgeSI  -0.595  0.588
## AgeA   -0.625  0.629  0.899
## Sex.fM -0.188  0.050  0.096  0.024
```

Summary

After accounting for variation across sampling country, there is a significant effect of Age and Sex in predicting LBV neutralization. The effect of sex is marginal, with males less likely to be seropositive.

1. Zuur, A., Ieno, E. N., Walker, N., Saveliev, A. A. & Smith, G. M. *Mixed Effects Models and Extensions in Ecology with R*. (Springer Science & Business Media, 2009).

Supplementary Table 2: Dam-pup pair serological titres

Date	Country	NiV ln(MFI)		LBV mFAVN Reciprocal titre	
		Dam	Pup	Dam	Pup
24/05/10	Bioko	2.3	2.4	9	15.59
25/05/10	Bioko	0.3	0.6	5.2	5.2
25/05/10	Bioko	1.5	1.4	9	9
25/05/10	Bioko	1.1	0.9	5.2	5.2
27/05/10	Bioko	2.6	3.4	5.2	5.2
27/05/10	Bioko	1.5	3.4	5.2	5.2
27/05/10	Bioko	1.8	2.5	5.2	5.2
31/03/10	São Tomé	3	3.6	9	81
31/03/10	São Tomé	3.4	3.9	5.2	5.2
31/03/10	São Tomé	2.3	2.7	5.2	9
31/03/10	São Tomé	2	1.8	46.77	27
31/03/10	São Tomé	1.4	1.8	81	81
31/03/10	São Tomé	2.5	2.7	9	46.77
3/04/10	São Tomé	3.7	3.8	5.2	5.2
3/04/10	São Tomé	1.2	1.5	81	243
3/04/10	São Tomé	2.6	3.4	5.2	5.2

Supplementary Table 3: Transition rates for the MSIRS model

Event	Transition	Transition rate
Birth of susceptible	$(M,S,I,R) \rightarrow (M,S+1,I,R)$	$B(t) * (S+I)$
Birth of maternally immune	$(M,S,I,R) \rightarrow (M+1,S,I,R)$	$B(t) * (R)$
Death of susceptible	$(M,S,I,R) \rightarrow (M,S-1,I,R)$	$m\ S$
Death of maternally immune	$(M,S,I,R) \rightarrow (M-1,S,I,R)$	$m\ M$
Death of infected	$(M,S,I,R) \rightarrow (M,S,I-1,R)$	$m\ I$
Death of recovered	$(M,S,I,R) \rightarrow (M,S,I,R-1)$	$M\ R$
Infection	$(M,S,I,R) \rightarrow (M,S-1,I+1,R)$	$\beta\ S\ I\ /\ (M+S+I+R)$
Recovery	$(M,S,I,R) \rightarrow (M,S,I,R+1)$	$\gamma\ I$
Loss of maternal immunity	$(M,S,I,R) \rightarrow (M-1,S+1,I,R)$	$\eta\ M$
Loss of acquired immunity	$(M,S,I,R) \rightarrow (M,S+1,I,R-1)$	$\zeta\ R$

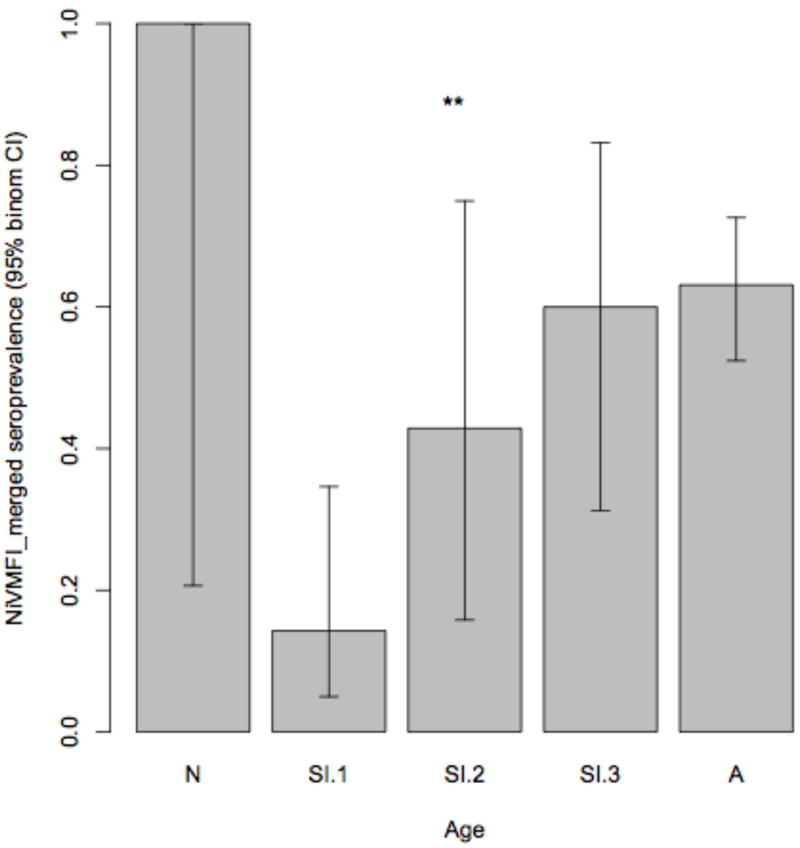

Supplementary Figure 1. Age-class seroprevalence in Annobón. Teeth were not collected in Annobón, however by sampling individuals at different time points over multiple years, sexually immature individuals could be further classified into 6 month age classes (SI.1 (6 - <12 months), SI.2 (12 - <18 months), SI.3 (18 - <24 months). No juveniles were sampled. Although sample sizes are small, age-class seroprevalence in Annobón is further supportive of active, endemic henipavirus transmission within sexually immature individuals. Insufficient seropositive individuals were detected to assess age-class seroprevalence for LBV on Annobón.

Supplementary Text 3: Force of Infection Models

```
library(powell)
library(tidyverse)
library(GGally)
library(doParallel)
registerDoParallel(cores=8)

# ======================== Likelihood function =======================
replace.par <- function(x,rep)
{
  if(length(rep)==0) return(x)
  pos <- sapply(names(rep),function(n) which(names(x)==n))
  replace(x,pos,rep)
}

# Expected seroprevalence at age a, assuming system at endemic equilibrium.
# MSIRS model
prev <- function(a,par) with(par,{
  exp(-a*(ri+lambda)) *
    (p*(ri+lambda)*(exp(a*(ri-rm+lambda))*(ri-rm)+lambda) +
    (exp(a*(ri+lambda))-1)*lambda*(lambda+ri-rm))/
    ((ri+lambda)*(ri-rm+lambda))
})

MSIRS.LL <- function(par, data){
  sum((data %>% mutate(P=dbinom(Pos,N,prev(Age,as.list(par)),log = T)))$P,na.rm = T)
}

# ========================= Calculate R0 ==============================
# Average life span in years
eidolon.LS <- 4.5

eidolon.R0 <- function(mle,L=eidolon.LS){
  d <- 1/L
  with(as.list(mle),{
    (d+ri+lambda)/(d+ri)/(1-p*d/(d+rm))
  })
}

# ======================= Import datasets ============================
load("HNV_LBV.RData")
HNV.table <- HNV.data %>% group_by(Age) %>% summarise(Pos=sum(Sero),N=n())
LBV.table <- LBV.data %>% group_by(Age) %>% summarise(Pos=sum(Sero),N=n())
```

Henipavirus

Data

```
ggplot(HNV.table, aes(x=Age, y=N)) + geom_bar(stat="identity") + ggtitle("Supplementary Figure 2
", subtitle = "Number of bats tested for Henipavirus antibodies by age (red bars show positives)
") + geom_bar(aes(y=Pos),fill="red",stat="identity")
```

Supplementary Figure 2: Number of bats tested for Henipavirus antibodies by age (red bars sh ow positives)

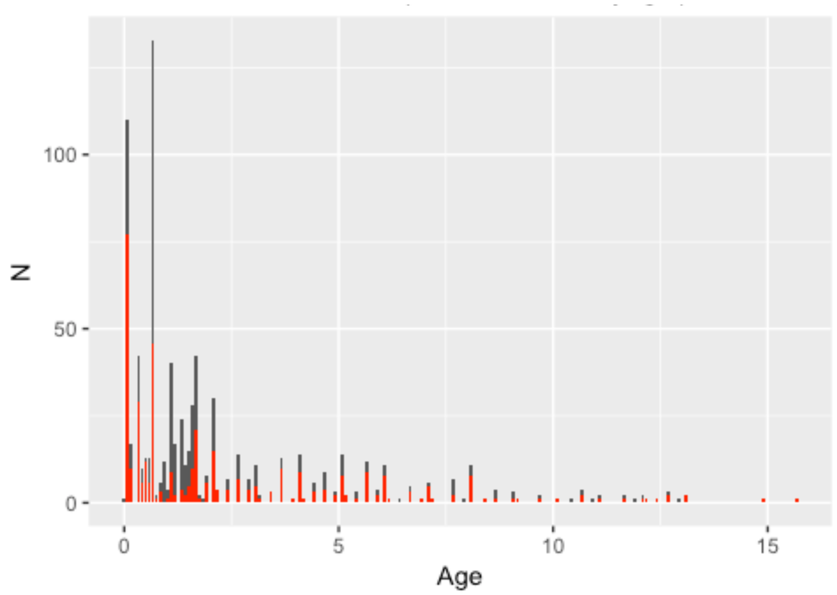

Model comparison

First, we compare the models with lifelong or waning immunity, using Akaike's Information Criterion (AIC).

```
HNV.par.0 <- c(p=0.5,lambda=2,ri=0.1,rm=2)

# ----------- Run the MLE ---------------------
HNV.mle.all <- powell(HNV.par.0, function(par){
  if(min(par)<0 | par[1]>1) return(1E9)
  names(par) <- names(HNV.par.0)
  ll <- MSIRS.LL(par,HNV.table)
  if(is.finite(ll)) -ll else 1E9
}, control=list(trace=1))
```

Model with waning immunity:

```
round(c(HNV.mle.all$par, LL = -HNV.mle.all$value, AIC = 2*HNV.mle.all$value + 2*length(HNV.mle.a
ll$par)),3)

      p   lambda       ri       rm       LL      AIC
  0.829    0.436    0.243    1.788 -115.792  239.584

HNV.par.1 <- c(p=0.5,lambda=0.5,rm=2)

# ----------- Run the MLE ---------------------
HNV.mle.1 <- powell(HNV.par.1, function(par){
  if(min(par)<0 | par[1]>1) return(1E9)
  names(par) <- names(HNV.par.1)
  ll <- MSIRS.LL(c(par,ri=0),HNV.table)
  if(is.finite(ll)) -ll else 1E9
}, control=list(trace=1))
```

Model with lifelong immunity:

```
round(c(HNV.mle.1$par, LL = -HNV.mle.1$value, AIC = 2*HNV.mle.1$value + 2*length(HNV.mle.1$par)),
3)

      p   lambda       rm       LL      AIC
  0.747    0.193    1.085 -133.665  273.330
```

Conclusion: Acquired immunity to Nipah is not life long (ΔAIC>10).

Parameter estimates and bootstrap confidence intervals

```
# ------------ Non-parametric bootstrap ----------------
N.BS <- 1000

HNV.mle.all.bs <- foreach(1:N.BS, .combine=rbind) %dopar% {
  bs.table <- HNV.data %>% sample_frac(replace=T) %>% group_by(Age) %>% summarise(Pos=sum(Sero),
N=n())
  mle <- powell(HNV.par.0, function(par){
    if(min(par)<0 | par[1]>1) return(1E9)
    names(par) <- names(HNV.par.0)
    ll <- MSIRS.LL(par,bs.table)
    if(is.finite(ll)) -ll else 1E9
  }, control=list(trace=1))
  c(mle$par, R0 = eidolon.R0(mle$par), LL=-mle$val,convergence=as.numeric(mle$convergence))
}

# Filter out failed convergence
HNV.mle.bs <- HNV.mle.all.bs %>% tbl_df %>% filter(convergence<1 & LL > -1E8)

# Confidence intervals
HNV.mle.bs.low <- HNV.mle.bs %>% summarise_at(1:5,quantile,probs=0.025) %>% round(3)
HNV.mle.bs.hi <- HNV.mle.bs %>% summarise_at(1:5,quantile,probs=0.975) %>% round(3)

HNV.mle.bs.CI <- rbind(HNV.mle.bs.low,HNV.mle.bs.hi) %>% mutate(mean.imm=round(1/lambda,1), mean.
ac=round(1/ri,1),mean.mat=round(1/rm,2))
```

Parameter estimates expressed as mean durations, with 95% CI:

- mean duration of maternal immunity (M -> S): 6.71 months (5.4, 8.4)
- mean time to acquire immunity (S -> R): 2.29 years (1.8, 3)
- mean duration of acquired immunity (R -> S): 4.12 years (2.7, 7.6)

Henipavirus R_0: 2.13 (1.96, 2.35).

Lagos Bat Virus

Data

```
ggplot(LBV.table, aes(x=Age, y=N)) + geom_bar(stat="identity") + ggtitle("Supplementary Figure 3
", subtitle = "Number of bats tested for LBV antibodies by age (red bars show positives)") + geo
m_bar(aes(y=Pos),fill="red",stat="identity")
```

Supplementary Figure 3: Number of bats tested for LBV antibodies by age (red bars show pos itives)

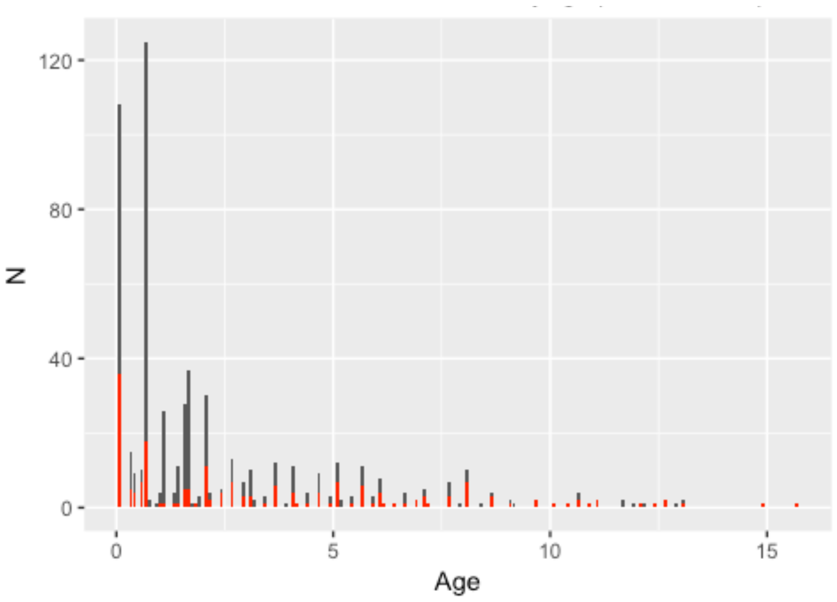

Model comparison

First, we compare the models with lifelong or waning immunity, using Akaike's Information Criterion (AIC).

```
LBV.par.0 <- c(p=0.5,lambda=2,ri=0.1,rm=2)

# ----------- Run the MLE ---------------------
LBV.mle.all <- powell(LBV.par.0, function(par){
  if(min(par)<0 | par[1]>1) return(1E9)
  names(par) <- names(LBV.par.0)
  ll <- MSIRS.LL(par,LBV.table)
  if(is.finite(ll)) -ll else 1E9
}, control=list(trace=1))
```

Model with waning immunity:

```
round(c(LBV.mle.all$par, LL = -LBV.mle.all$value, AIC = 2*LBV.mle.all$value + 2*length(LBV.mle.a
ll$par)),3)

      p  lambda      ri      rm      LL     AIC
  0.414   0.174   0.081   2.192 -88.770 185.539

LBV.par.1 <- c(p=0.5,lambda=0.5,rm=2)

# ----------- Run the MLE ---------------------
LBV.mle.1 <- powell(LBV.par.1, function(par){
  if(min(par)<0 | par[1]>1) return(1E9)
  names(par) <- names(LBV.par.1)
  ll <- MSIRS.LL(c(par,ri=0),LBV.table)
  if(is.finite(ll)) -ll else 1E9
}, control=list(trace=1))
```

Model with lifelong immunity:

```
round(c(LBV.mle.1$par, LL = -LBV.mle.1$value, AIC = 2*LBV.mle.1$value + 2*length(LBV.mle.1$par)),
3)

      p  lambda      rm      LL     AIC
  0.402   0.130   1.881 -90.908 187.815
```

Conclusion: Marginal support for life long immunity (ΔAIC=2.3).

Parameter estimates and bootstrap confidence intervals

```
# ------------ Non-parametric bootstrap ----------------
N.BS <- 1000

LBV.mle.all.bs <- foreach(1:N.BS, .combine=rbind) %dopar% {
  bs.table <- LBV.data %>% sample_frac(replace=T) %>% group_by(Age) %>% summarise(Pos=sum(Sero),
N=n())
  mle <- powell(LBV.par.0, function(par){
    if(min(par)<0 | par[1]>1) return(1E9)
    names(par) <- names(LBV.par.0)
    ll <- MSIRS.LL(par,bs.table)
    if(is.finite(ll)) -ll else 1E9
  }, control=list(trace=1))
  c(mle$par, R0 = eidolon.R0(mle$par), LL=-mle$val,convergence=as.numeric(mle$convergence))
}

# Filter out failed convergence
LBV.mle.bs <- LBV.mle.all.bs %>% tbl_df %>% filter(convergence<1 & LL > -1E8)

# Confidence intervals
LBV.mle.bs.low <- LBV.mle.bs %>% summarise_at(1:5,quantile,probs=0.025) %>% round(3)
LBV.mle.bs.hi <- LBV.mle.bs %>% summarise_at(1:5,quantile,probs=0.975) %>% round(3)

LBV.mle.bs.CI <- rbind(LBV.mle.bs.low,LBV.mle.bs.hi) %>% mutate(mean.imm=round(1/lambda,1), mean.
ac=round(1/ri,1),mean.mat=round(1/rm,2))
```

Parameter estimates expressed as mean durations, with 95% CI:

- mean duration of maternal immunity (M -> S): 5.47 months (3.6, 8.76)
- mean time to acquire immunity (S -> R): 5.75 years (4.1, 7.9)
- mean duration of acquired immunity (R -> S): 12.27 years (5.2,)

Henipavirus R_0: 1.64 (1.53, 1.77).

Visualisation of bootstrap estimates

```
All.mle.bs <- bind_rows(
  HNV.mle.bs %>% mutate(Virus="HNV"),
  LBV.mle.bs %>% mutate(Virus="LBV")
)

All.mle.bs %>% ggpairs(mapping = aes(colour=Virus), columns=c(1:5),lower=list(continuous="densit
y")) + ggtitle("Supplementary Figure 4", subtitle = "Density plots of bootstrap estimates for He
nipavirus (HNV, red) and Lagos bat virus (LBV, blue)")
```

Supplementary Figure 4: Density plots of bootstrap estimates for Henipavirus (HNV, red) and Lagos bat virus (blue)

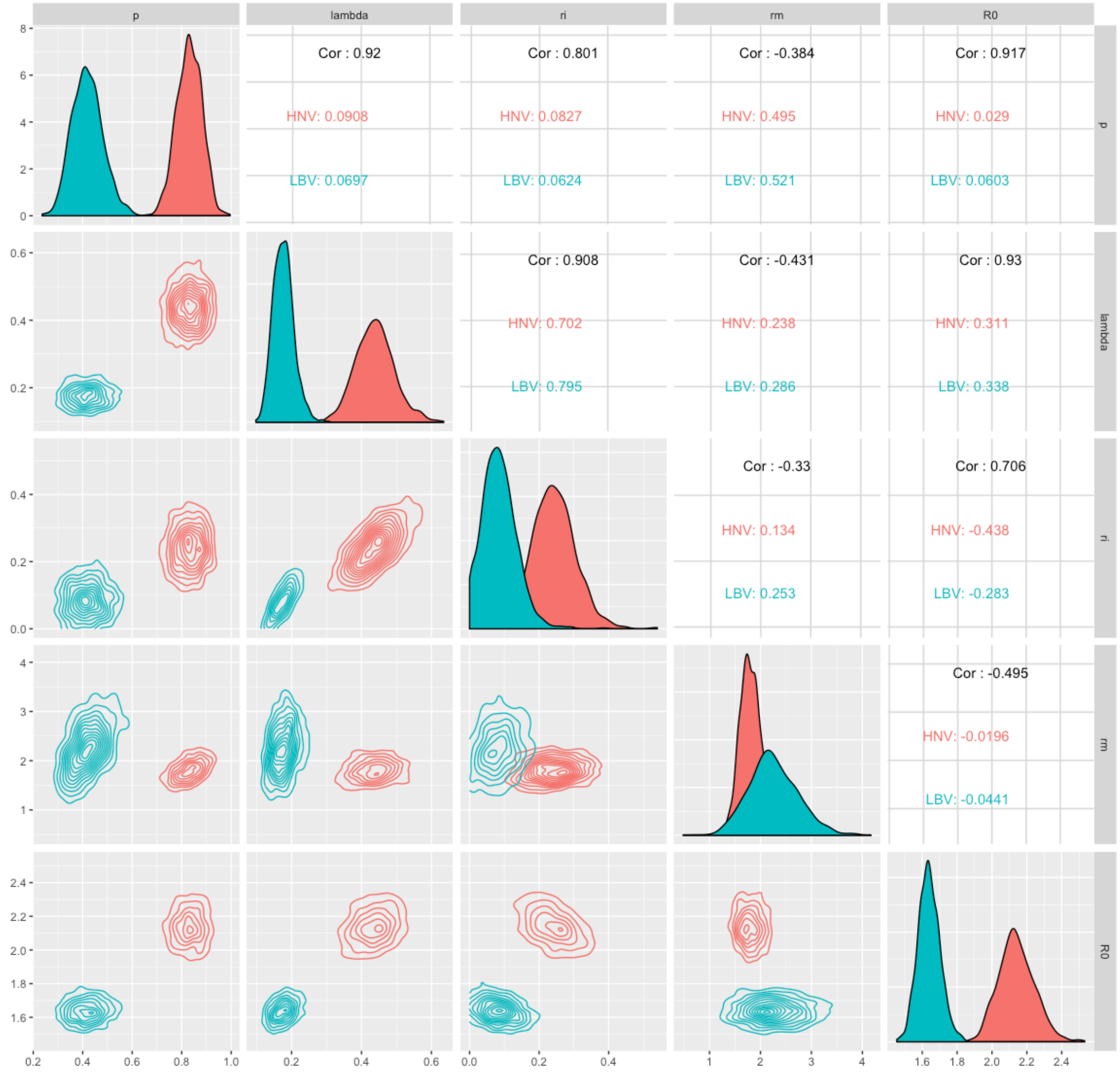

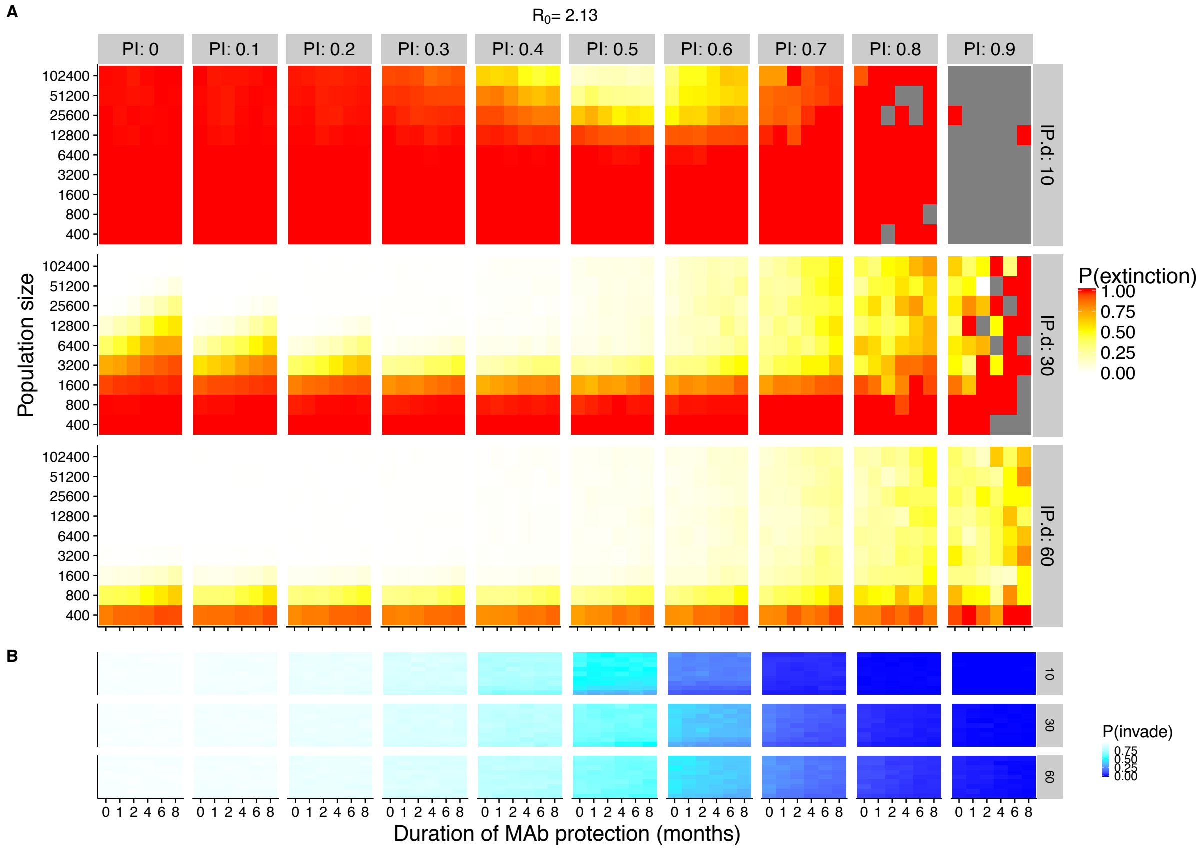

Supplementary Figure 5 Effect of the duration of maternal antibody protection (in months, MAb), proportion of acquire population immunity (PI) and infectious period (in days, IP.d) on persistence of infection at various population sizes.

A) Probability of pathogen extinction within 10 years of introduction (conditional on successful introduction) as a function of population size according to the colour scale shown. The probability of successful invasion decreased with increasing population immunity, meaning that for very high values of population immunity, none of the 1000 simulations were able to invade for some parameter values (grey areas in figure).

B) Probability of successful pathogen invasion as a function of population size according to the colour scale shown.

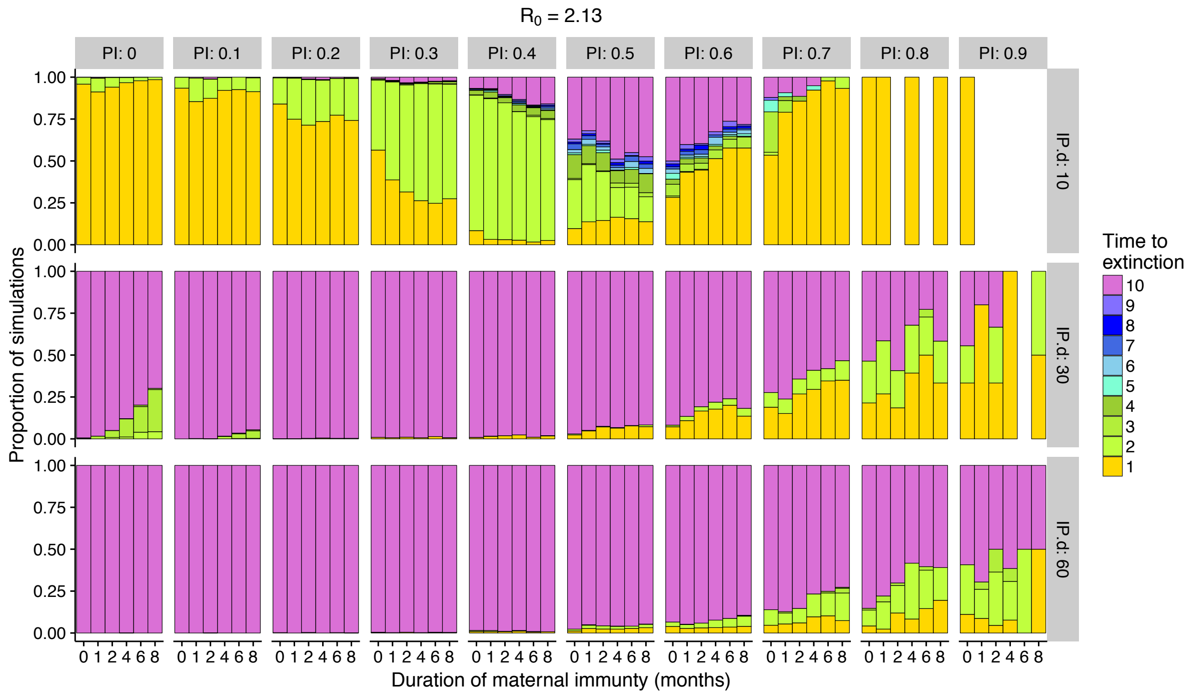

Supplementary Figure 6 – Effect of the duration of maternal antibody protection (in months, MAb), proportion of acquired population immunity (PI) and infectious period (in days, IP.d) on persistence of infection. Stacked histograms show time to pathogen extinction (conditional on successful invasion) in series of 1000 stochastic simulations run for 10 years in a population of 25,600 individuals. Parameter values: mean lifespan = 4.5 years, s = 14.3, τ = 0.25, R_0 = *2.13*.

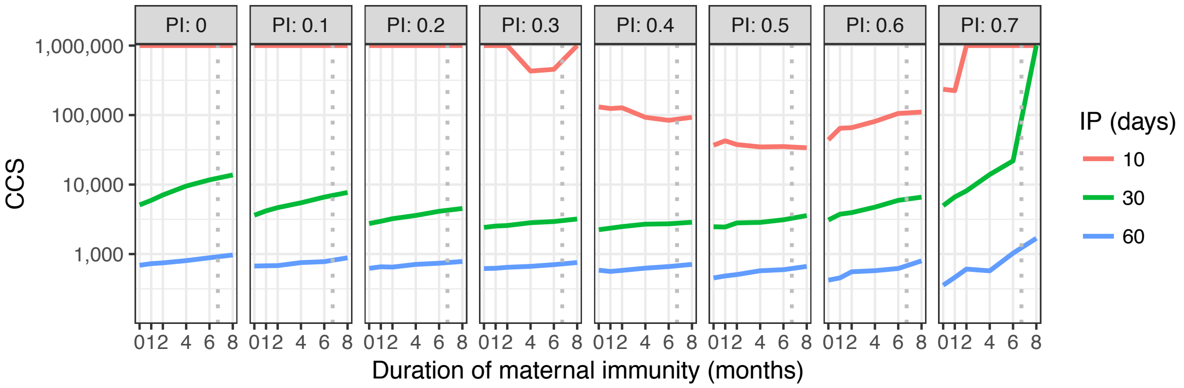

Supplementary Figure 7 – Effect of the duration of maternal antibody protection (in months, MAb), proportion of acquired population immunity (PI) and infectious period (in days, IP) on the population size for which successful of invasion and persistence of infection is more probable than not (critical community size, CCS). Grey dotted lines show the mean duration of maternal immunity, as calculated in the age-specific immunity model (henipaviruses = 6.7 months). For some sets of parameter values, probability of invasion was very low (Supplementary Figure 2), resulting in low precision of the CCS estimate, as demonstrated by the jagged lines. Plots for PI>0.7 are not shown for this reason. Parameter values: mean lifespan = 4.5 years, s = 14.3, τ = 0.25, R_0 = 2.13.

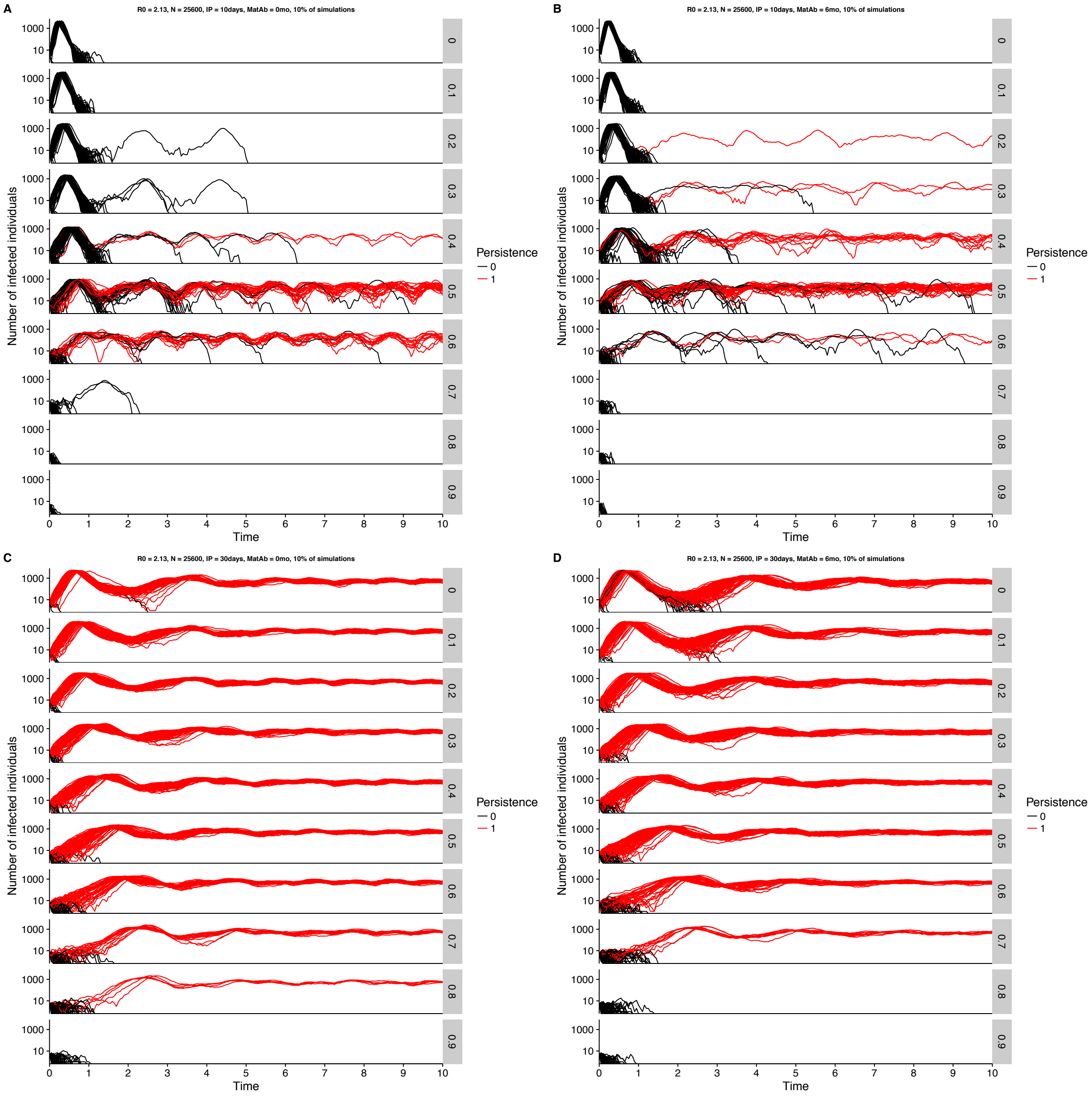

Supplementary Figure 8 – Subset of simulations (10%) from the MSIRS model showing the non-monotonic effect of population immunity (rows, 0-0.9) on epidemic and endemic fadeout for viruses with an infectious period of 10 (A, B) or 30 days (C, D) and a mean maternal antibody duration of 0 months (A, C) or 6 months (B, D). The x-axis shows time in years and y-axis shows the number of infected individuals. Parameter values are the same as those shown in the three columns in Figure 4: mean lifespan = 4.5 years, s = 14.3, τ = 0.25, R_0 = 2.13. Mean maternal antibody duration, Infectious period and population size shown above each plot.

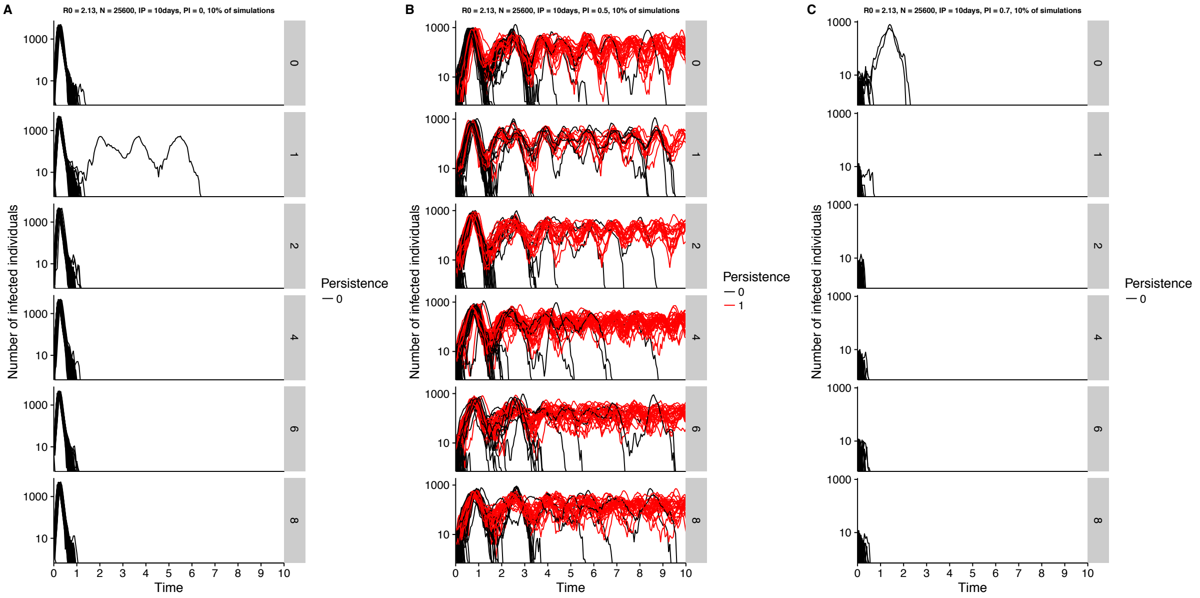

Supplementary Figure 9 – Subset of simulations (10%) from the MSIRS model showing the effect of maternal immunity (rows, 0-8 months) on epidemic and endemic fadeout for viruses with an infectious period of 10 days and a acquired population immunity of 0 months (A), 0.5 (B) or 0.7 (C). The x-axis shows time in years and y-axis shows the number of infected individuals. Parameter values are the same as those shown in the three columns in Figure 4: mean lifespan = 4.5 years, s = 14.3, τ = 0.25, R_0 = 2.13. Mean maternal antibody duration, Infectious period and population size shown above each plot.

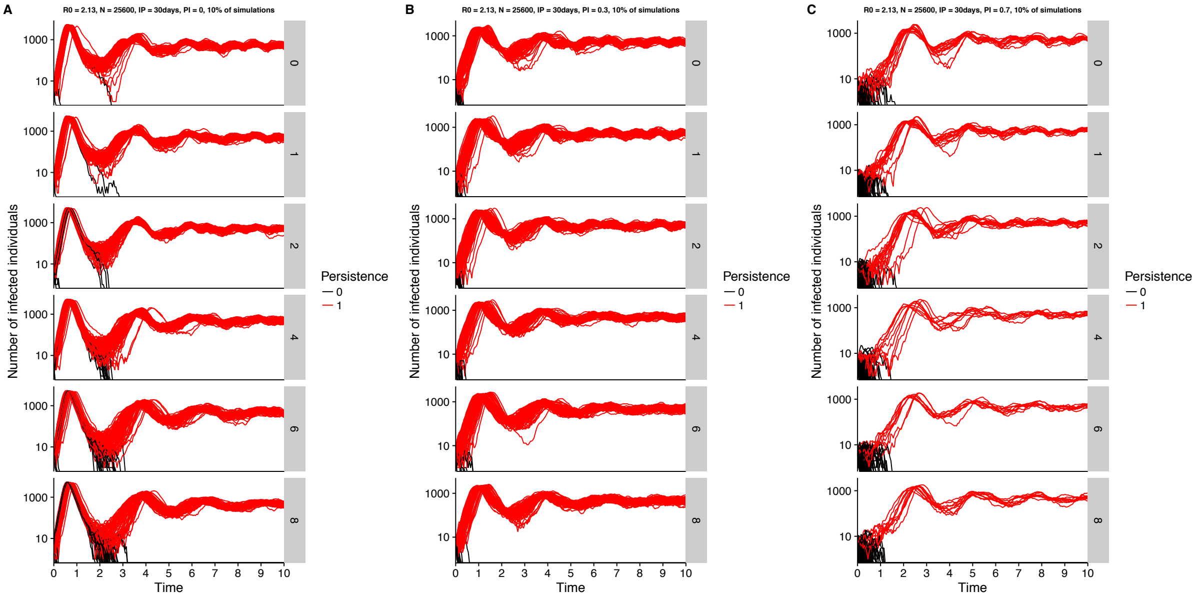

Supplementary Figure 10 – Subset of simulations (10%) from the MSIRS model showing the effect of maternal immunity (rows, 0-8 months) on epidemic and endemic fadeout for viruses with an infectious period of 30 days and a acquired population immunity of 0 months (A), 0.5 (B) or 0.7 (C). The x-axis shows time in years and y-axis shows the number of infected individuals. Parameter values are the same as those shown in the three columns in Figure 4: mean lifespan = 4.5 years, s = 14.3, τ = 0.25, R_0 = 2.13. Mean maternal antibody duration, Infectious period and population size shown above each plot.

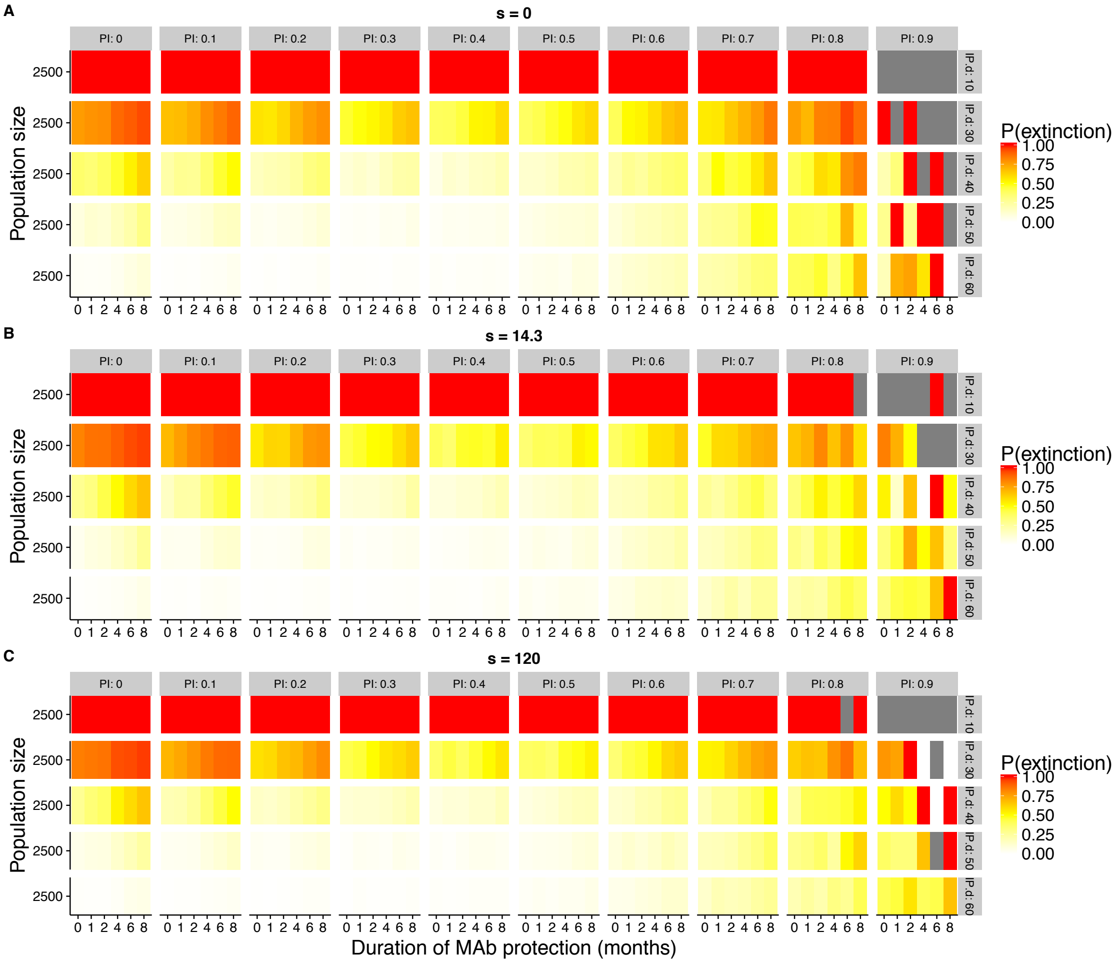

Supplementary Figure 11 Effect of the duration of maternal antibody protection (in months, MAb), proportion of acquire population immunity (PI) and infectious period (in days, IP.d) on persistence of infection at a population size of 2500 individuals (as estimated for *Eidolon* helvum on Annobón island, Equatorial Guinea). Probability of pathogen extinction within 10 years of introduction (conditional on successful introduction) as a function of population size according to the colour scale shown. The probability of successful invasion decreased with increasing population immunity, meaning that for very high values of population immunity, none of the 1000 simulations were able to invade for some parameter values (grey areas in figure).

A: s = 0 (constant births), B: s = 14.3 (estimated birth pulse duration for *E. helvum*, representing 95% of births occurring within 3.2 months), C) s = 120 (representing 95% of births occurring within 1.2 months). For each combination of parameter values, 1000 stochastic simulations were run. Parameter values: mean lifespan = 4.5 years, s = 14.3, τ = 0.25, R_0 = 2.13.

In B), following introduction in a naive population (PI = 0) comprising 2500 individuals or less, persistence is likely (>50% probability) for viruses with infectious period ≥40 days (IP.d = 40), regardless of maternal antibody (MatAb) protection. If the duration of MatAb is ≥6 months, then an infectious period of ≥50 days is likely required for persistence. In a non-naïve population with intermediate seroprevalences (e.g. PI = 0.4 or 0.5), extinction is only likely for infectious periods ≤ 10days.

References:

Allerson, M., Deen, J., Detmer, S. E., Gramer, M. R., Joo, H. S., Romagosa, A., & Torremorell, M. (2013). The impact of maternally derived immunity on influenza A virus transmission in neonatal pig populations. *Vaccine*, *31*(3), 500–505. http://doi.org/10.1016/j.vaccine.2012.11.023

Amman, B. R., Carroll, S. A., Reed, Z. D., Sealy, T. K., Balinandi, S., Swanepoel, R., et al. (2012). Seasonal Pulses of Marburg Virus Circulation in Juvenile Rousettus aegyptiacus Bats Coincide with Periods of Increased Risk of Human Infection. *PLoS Pathogens*, *8*(10), e1002877. http://doi.org/10.1371/journal.ppat.1002877

Baker, K. S., Suu-Ire, R., Barr, J. A., Hayman, D. T. S., Broder, C. C., Horton, D. L., et al. (2013). Viral antibody dynamics in a chiropteran host. *Journal of Animal Ecology*, *83*(2), 415–428. http://doi.org/10.1111/1365-2656.12153

Bichara, D., Iggidr, A., & Sallet, G. (2013). Global analysis of multi-strains SIS, SIR and MSIR epidemic models. *Journal of Applied Mathematics and Computing*, *44*(1-2), 273–292. http://doi.org/10.1007/s12190-013-0693-x

Cador, C., Rose, N., Willem, L., & Andraud, M. (2016). Maternally Derived Immunity Extends Swine Influenza A Virus Persistence within Farrow-to-Finish Pig Farms: Insights from a Stochastic Event-Driven Metapopulation Model. *PLoS ONE*, *11*(9), e0163672. http://doi.org/10.1371/journal.pone.0163672

Chapman, J. D. (2010). *Mathematical Modelling of Epidemic Systems Influenced by Maternal Antibodies and Public Health Intervention*. wrap.warwick.ac.uk.

Constantine, D. G., Tierkel, E. S., Kleckner, M. D., & Hawkins, D. M. (1968). Rabies in New Mexico cavern bats. *Public Health Reports*, *83*(4), 303–316.

Epstein, J. H., Baker, M. L., Zambrana-Torrelio, C., Middleton, D., Barr, J. A., DuBovi, E., et al. (2013). Duration of Maternal Antibodies against Canine Distemper Virus and Hendra Virus in Pteropid Bats. *PLoS ONE*, *8*(6). http://doi.org/10.1371/journal.pone.0067584

Field, H. E. (2005). *The ecology of Hendra virus and Australian bat lyssavirus. PhD Thesis*. University of Queensland.

Fouchet, D., Marchandeau, S., Bahi-Jaber, N., & Pontier, D. (2007). The role of maternal antibodies in the emergence of severe disease as a result of fragmentation. *Journal of the Royal Society Interface*, *4*(14), 479–489. http://doi.org/10.1098/rsif.2006.0189

Garnier, R., Gandon, S., Harding, K. C., & Boulinier, T. (2014). Length of intervals between epidemics: evaluating the influence of maternal transfer of immunity. *Ecology and Evolution*, *4*(5), 568–575.

Homwong, N. (2016). *Bayesian Modeling of Within-Herd Transmission Dynamics of Swine Influenza Virus*. conservancy.umn.edu.

Kallio, E. R., Begon, M., Henttonen, H., Koskela, E., Mappes, T., Vaheri, A., & Vapalahti, O. (2010). Hantavirus infections in fluctuating host populations: the role of maternal antibodies. *Proceedings of the Royal Society B: Biological Sciences*, *277*(1701), 3783–3791. http://doi.org/10.1098/rspb.2010.1022

Philbey, A. W., Kirkland, P. D., Ross, A. D., Field, H. E., Srivastava, M., Davis, R. J., & Love, R. J. (2008). Infection with Menangle virus in flying foxes (Pteropus spp.) in Australia. *Australian Veterinary Journal*, *86*(11), 449–454. http://doi.org/10.1111/j.1751-0813.2008.00361.x

Plowright, R. K., Field, H. E., Smith, C., Divljan, A., Palmer, C., Tabor, G., et al. (2008). Reproduction and nutritional stress are risk factors for Hendra virus

infection in little red flying foxes (Pteropus scapulatus). *Proceedings of the Royal Society B: Biological Sciences*, *275*(1636), 861–869. http://doi.org/10.1098/rspb.2007.1260

Plowright, R. K., Foley, P., Field, H. E., Dobson, A. P., Foley, J. E., Eby, P., & Daszak, P. (2011). Urban habituation, ecological connectivity and epidemic dampening: the emergence of Hendra virus from flying foxes (*Pteropus* spp.). *Proceedings of the Royal Society B: Biological Sciences*, *278*(1725), 3703–3712. http://doi.org/10.1098/rspb.2011.0522

Price, J. L., & Everard, C. O. R. (1977). RABIES VIRUS AND ANTIBODY IN BATS IN GRENADA AND TRINIDAD. *Journal of Wildlife Diseases*, *13*(2), 131–134.

Pulliam, J. R. C., Epstein, J. H., Dushoff, J., Rahman, S. A., Bunning, M., Jamaluddin, A. A., et al. (2012). Agricultural intensification, priming for persistence and the emergence of Nipah virus: a lethal bat-borne zoonosis. *Journal of the Royal Society Interface*, *9*(66), 89–101. http://doi.org/10.1098/rsif.2011.0223

Shankar, V., Bowen, R. A., Davis, A. D., Rupprecht, C. E., & O'Shea, T. J. (2004). Rabies in a captive colony of big brown bats (Eptesicus fuscus). *Journal of Wildlife Diseases*, *40*(3), 403–413.

Sohayati, A. R., Hassan, L., Sharifah, S. H., Lazarus, K., Zaini, C. M., Epstein, J. H., et al. (2011). Evidence for Nipah virus recrudescence and serological patterns of captive *Pteropus vampyrus*. *Epidemiology and Infection*, *139*(10), 1570–1579. http://doi.org/10.1017/S0950268811000550

Steece, R., & Altenbach, J. S. (1989). Prevalence of rabies specific antibodies in the Mexican free-tailed bat (*Tadarida brasiliensis mexicana*) at Lava Cave, New Mexico. *Journal of Wildlife Diseases*, *25*(4), 490–496.

Wells, K., Brook, B. W., Lacy, R. C., Mutze, G. J., Peacock, D. E., Sinclair, R. G., et al. (2015). Timing and severity of immunizing diseases in rabbits is controlled by seasonal matching of host and pathogen dynamics. *Journal of the Royal Society Interface*, *12*(103), 20141184–20141184. http://doi.org/10.1098/rsif.2014.1184

Zuur, A., Ieno, E. N., Walker, N., Saveliev, A. A., & Smith, G. M. (2009). Mixed Effects Models and Extensions in Ecology with R. Springer
